# *H. Pylori*‐Facilitated TERT/Wnt/β‐Catenin Triggers Spasmolytic Polypeptide‐Expressing Metaplasia and Oxyntic Atrophy

**DOI:** 10.1002/advs.202401227

**Published:** 2024-11-25

**Authors:** Lijiao He, Xiao Zhang, Shengwei Zhang, Yi Wang, Weichao Hu, Jie Li, Yunyi Liu, Yu Liao, Xue Peng, Jianjun Li, Haiyan Zhao, Liting Wang, Yang‐Fan Lv, Chang‐Jiang Hu, Shi‐Ming Yang

**Affiliations:** ^1^ Department of Gastroenterology The Second Affiliated Hospital of Army Medical University Chongqing 400037 China; ^2^ Cancer Center of Daping Hospital Army Medical University Chongqing 400000 China; ^3^ Department of Gastroenterology The 987th Hospital of the Joint Logistics Support Force of the People's Liberation Army of China, Baoji Shaanxi 721000 China; ^4^ Biological Science Research Center Southwest University Chongqing 400715 China; ^5^ Central Laboratory Army Medical University Chongqing 400038 China; ^6^ Department of Pathology The Second Affiliated Hospital of Army Medical University Chongqing 400037 China

**Keywords:** CagA, H. pylori infection, nitazoxanide, SPEM, TERT

## Abstract

Persistent *H. pylori* infection triggers the repair program of the mucosa, such as spasmolytic polypeptide‐expressing metaplasia (SPEM). However, the mechanism underlying the initiation of SPEM in gastric tissues by *H. pylori* remains unclear. Here, an increase in telomerase reverse transcriptase (TERT) protein expression is observed in chief cells upon infection with *cagA*‐positive *H. pylori*. *Tert* knockout significantly ameliorated *H. pylori*‐induced SPEM and single‐cell RNA sequencing demonstrated that the Wnt/β‐Catenin pathway is suppressed in gastric cells with *Tert* knockout. Mechanism study revealed that CagA elevated TERT abundance by disrupting the interaction between TERT and its novel E3 ligase, SYVN1. Interestingly, Nitazoxanide effectively relieved SPEM via inhibition of the Wnt/β‐Catenin signaling in vivo. This results clarified the mechanism underlying which CagA activated the TERT/Wnt/β‐Catenin pathway, thus promoting the dedifferentiation of chief cells and the occurrence of SPEM in gastric mucosa. This highlights a molecular basis for targeting CagA‐activated Wnt signaling in chief cells for the treatment of gastric precancerous lesions.

## Graphical abstract

*H. pylori* infection leads repair program of the mucosa, like spasmolytic polypeptide‐expressing metaplasia (SPEM). The toxic factors of *H. pylori*, CagA, increased the level of TERT by disrupting the interaction between TERT and its E3 ligase, SYVN1. TERT activation of the Wnt signaling pathway leads to the occurrence of SPEM. Nitazoxanide alleviated this process induced by *H. pylori* infection.

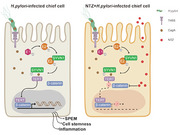

## 1 Introduction

As the primary carcinogen of gastric cancer, *Helicobacter pylori* (*H. pylori*) is widely accepted to be associated with mucosal barrier disruption and triggers the initiation of the Correa model, which involves chronic non‐atrophic gastritis (CG), atrophic gastritis (AG), intestinal metaplasia (IM) and, ultimately, gastric cancer.^[^ ^1^ ^]^

*H. pylori* colonizes the surface of gastric mucosal epithelium and causes gastric mucosal lesions by secreting toxic factors. CagA is widely recognized as the primary virulence determinant of *H. pylori*. It is delivered into the epithelium cell cytoplasm through the type IV secretion system. It exerts its pathogenic effects by modulating numerous signaling pathways, including Wnt/β‐Catenin, Ras/MEK/ERK, PAR1B/BRCA1/YAP and PI3K/AKT.^[^ ^2^, ^3^, ^4^, ^5^, ^6^, ^7^ ^]^ Persistent *H. pylori* infection leads to sustained inflammatory stimulation, triggering a response known as gastric gland metaplasia in the stomach. Prolonged chronic inflammation renders the metaplastic tissue susceptible to malignant transformation.^[^ ^8^, ^9^, ^10^ ^]^

The metaplasia of gastric mucosa can be classified into two types according to histopathology: IM and SPEM. SPEM is a common reprogramming process of various body organs responding to injury.^[^ ^11^ ^]^ In the stomach, SPEM is characterized by the expansion of tuft cells, hyperplasia of neck cells, formation of metaplasia cells, and the infiltration of M2 macrophages, eosinophils, and other immune cells in the corpus mucosa.^[^ ^12^, ^13^, ^14^, ^15^ ^]^ Mature chief cells have the potential to undergo transdifferentiation and transform into SPEM cells. When tissue damage occurs, a subset of mature differentiated chief cells has been shown to exhibit a redifferentiation capability in response to mucosal injury, as reported by several studies.^[^ ^12^, ^16^, ^17^, ^18^, ^19^, ^20^ ^]^

The development of SPEM is driven by a conserved molecular network. In the event of acute gastric injury, DDIT4 leads to extensive autophagy that breaks down the structure of differentiated cells via inhibiting mTORC1 expression in SPEM models induced by high‐dose tamoxifen.^[^ ^11^, ^21^ ^]^ In *H. pylori* chronic infection models, *H. pylori* colonizes and manipulates the Lgr5+ gland cells, leading to the up‐regulating expression of stem‐related genes and glandular hyperplasia.^[^ ^22^ ^]^ Further studies have demonstrated that lgr5+ stem cells are a subset of chief cells that expand to dedifferentiate into SPEM cells in response to injury.^[^ ^16^, ^23^ ^]^ Furthermore, a single study has suggested that the activation of Wnt‐signaling pathways occurred during the development of SPEM induced by stCldn18 deficiency.^[^ ^24^ ^]^ Another study has reported that SPO3, a Wnt‐signaling enhancer, regulates the differentiation of secretory cells in the stomach corpus gland, particularly toward parietal and chief cells.^[^ ^25^ ^]^ However, the specific molecular mechanism underlying the derivation of SPEM cells induced by chronic infection of *H. pylori* remains unclear.

Telomere reverse transcriptase (TERT) is an important component of telomerase, acting as the catalytic subunit in the reverse transcription to synthesize telomeric DNA. Recent studies have revealed that TERT has additional biological functions independent of its role in telomere length regulation.^[^ ^26^ ^]^ Specifically, it has been found that TERT can directly interact with transcription factors to serve as a coactivator, regulating the expression of downstream target genes in Wnt, NF‐κB and other signaling pathways.^[^ ^27^, ^28^ ^]^ Several studies have found a correlation between the expression of TERT and the high risk of IM, which is more likely to progress into gastric cancer.^[^ ^29^, ^30^ ^]^ However, despite these advancements, the detailed mechanism of action of TERT in IM remains poorly understood, warranting further investigation into its specific role in this context.

Here, we initiated the investigation of TERT's role in IM by assessing TERT protein levels in oxyntic mucosa biopsies from patients with intestinal metaplasia. The analysis uncovered a correlation between the expression of TERT in the gastric gland and *cagA*‐positive *H. pylori* infection, leading to the speculation of CagA‐inducing SPEM through TERT modulation. Subsequently, a *H. pylori*‐infected mouse model was established using the wild‐type and the *cagA* knockout *H. pylori*. The mechanism of TERT in CagA‐induced SPEM was further explored through single‐cell RNA sequencing and cellular molecular biology experiments. Additionally, we evaluated the potential of the drug nitazoxanide in the treatment of CagA‐induced SPEM.

## 2 Results

### 2.1 CagA‐Positive *H. Pylori* Infection Facilitates TERT Protein Expression in Human

**Figure 1:**
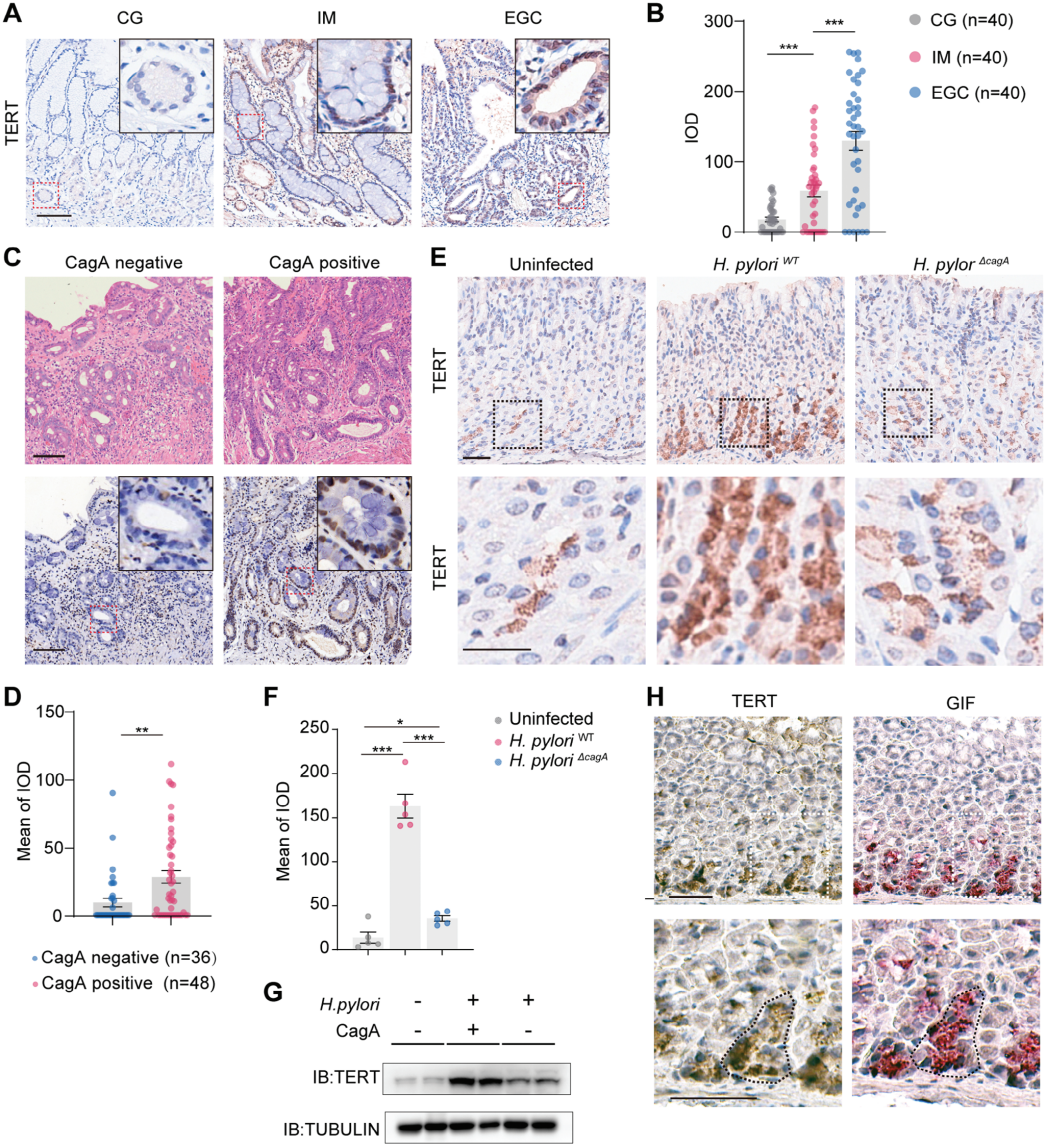
CagA‐positive *H. pylori* promotes the expression of TERT in the gastric mucosa of humans and mice. A,B) The immunohistochemical staining intensity of TERT in CG (*n* = 40), IM (*n* = 40) and EGC tissues (*n* = 40). Scale bar, 100 µm. C,D) The immunohistochemical staining intensity of TERT in the gastric IM tissue from anti‐CagA antibodies negative (*n* = 36) and positive (*n* = 48) patients with *H. pylori* infection. Scale bar, 200 µm. E,F) The immunohistochemical staining intensity of TERT on uninfected (*n* = 5), *H. pylori^WT^* infected (*n* = 5) and *H. pylori^ΔcagA^* infected mice (*n* = 5) gastric mucosa. G) Western blot analysis of TERT protein in mice gastric mucosa. H) Immunohistochemical staining of TERT and GIF in serial sections of gastric body mucosa from mice infected with *H. pylori^WT^*. Scale bar, 50 µm. *p*‐values: ^*^ *p* < .05; ^**^ *p* < .01; ^***^ *p* < .001. CG, chronic gastritis; IM, intestinal metaplasia; EGC, early gastric cancer; IOD, integrated option density. Errors bars represent mean ± SEM of biological replicates.

Different stages of gastric tissues, including 40 cases each of CG, IM, and early gastric cancer (EGC), were collected from the pathological specimen library of Xinqiao Hospital. Immunohistochemical staining showed TERT expression gradually increased IM and EGC, compared to CG (**Figure** **1**A,B). To investigate the role of TERT in intestinal metaplasia, oxyntic stomach tissues were collected from 105 CG patients and 115 IM patients, and the *H. pylori* infection status of the patients was examined (Figure S1A, Supporting Information). Patient characteristics were shown in Table S1 (Supporting Information). Immunofluorescence staining demonstrated a reduction in the number of parietal cells (H^+^K^+^ATPase^+^) and chief cells (PGC^+^) in IM tissue (Figure S1B, Supporting Information). Additionally, SPEM cell (PGC^+ ^GS II^+^) was identified in the IM gastric mucosa. Cell markers associated with IM were detected by quantitative real‐time PCR (qRT‐PCR). The results showed decreased expression of the parietal cell marker *ATP4B* and the chief cell marker *PGC* in IM tissues, along with a notable elevation in the IM marker *CDX1* (Figure S1C, Supporting Information). Furthermore, the markers *CD44v9* and *CFTR*, associated with SPEM cells, exhibited upregulation in IM patients.

The potential interplay between TERT and *H. pylori* was investigated by examining *H. pylori* infection in patients. Among 115 cases, 84 tested positive for *H. pylori* infection, and 48 of these individuals displayed anti‐CagA antibodies in their serum. TERT expression was significantly up‐regulated in the CagA‐positive *H. pylori*‐infected cases compared to the CagA‐negative cases (Figure 1C,D). Immunofluorescence analysis revealed a significant increase in SPEM proportion in the CagA‐positive group (Figure S1D,E, Supporting Information). The mRNA expression of *IL8*, *AXIN2*, *CD44v9* and *CFTR* were amplified in the CagA‐positive group (Figure S1F, Supporting Information). These observations led to the speculation that CagA stimulates the expression of TERT and exacerbates the development of SPEM in the human gastric mucosa. Thus, TERT likely plays a role in the development of SPEM during *H. pylori* infection.

### 2.2 *cagA*‐Positive *H. Pylori* Infection Promotes SPEM by Up‐Regulating TERT in Chief Cells

To further examine the potential impact of CagA on promoting SPEM through TERT regulation, the C57BL/6 mice were subjected to infection with *cagA*‐positive PMSS1 (*H. pylori^WT^*) or *cagA*‐negative PMSS1 (*H. pylori* ^*ΔcagA*^). Mice infected with *H. pylori^WT^* showed a higher presence of inflammatory cells at the bottom of gastric glands compared to those infected with *H. pylori* ^*ΔcagA*^, suggesting a stronger inflammation in *H. pylori^WT^*‐infected mice (Figure S2A, Supporting Information). *H. pylori* were detected infiltrating the deep region of gastric foveola using the Warthin‐Starry silver staining technique (Figure S2B, Supporting Information). The colonization of *H. pylori* was measured using probe‐PCR, but the outcome indicated no apparent association between CagA and *H. pylori* colonization (Figure S2C, Supporting Information). Furthermore, the gastric corpus glands of *H. pylori^WT^*‐infected mice exhibited a noteworthy rise in SPEM cells (GIF^+^ GS II^+^), while the number of chief cells (GIF^+^) decreased (Figure S2D, Supporting Information). Furthermore, we identified the SPEM region using the CD44v9 antibody. We discovered a marked reduction in the count of parietal cells (H^+^K^+^ATPase^+^) within the SPEM region of *H. pylori^WT^*‐infected mice (Figure S2E,F, Supporting Information). Notably, there were no significant alterations observed in neck cells (GS II+).

These results were also validated by qRT‐PCR. In comparison to the uninfected group, *Gif* was down‐regulated in *H. pylori^WT^* infected mice, coupled with an increased expression of SPEM markers, including *He4*, *Cd44v9*, *Dmbt1*, *Gkn3*, *Clu* and *Cftr* (Figure S2G, Supporting Information), which were dramatically alleviated in the *H. pylori* ^*ΔcagA*^‐infected mice. Immunohistochemistry and western blot showed that TERT was remarkably increased after *cagA*‐positive *H. pylori* infection (Figure 1E–G). Besides, serial paraffin sections indicated that TERT protein was mainly expressed in chief cells (GIF^+^) (Figure 1H). Collectively, these data suggest that TERT was up‐regulated in chief cells of *H. pylori^WT^*‐infected mice.

### 2.3 Single‐Cell RNA Sequencing Reveals That *Tert* Knockout Ameliorates CagA‐Induced SPEM

**Figure 2:**
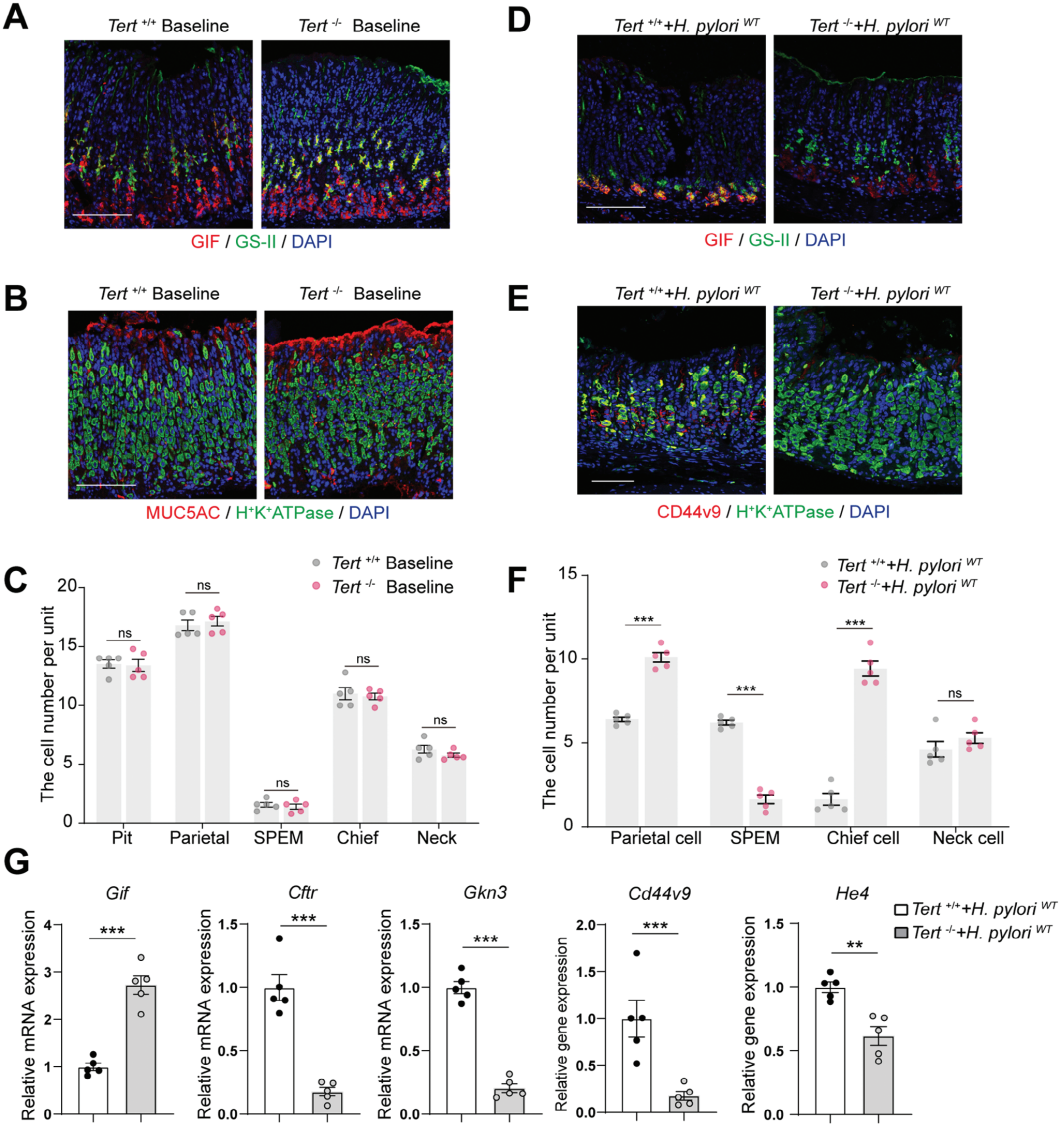
The occurrence of SPEM is ameliorated in *H. pylori*‐infected *Tert* ^−/−^ mice. A) Representative immunofluorescent staining of GIF (red color) and GS II (green color) at baseline level in *Tert^+/+^* and *Tert^− /−^* mice. Scale bar, 100 µm. B) Representative immunofluorescent staining of MUC5AC (red color) and H^+^K^+^ATPase (green color) at baseline level in *Tert^+/+^* and *Tert^−/−^* mice. Scale bar, 100 µm. C) Quantification of cell numbers per gland unit from panels A and B. Each group had 5 mice. Each data point represents the mean number of each type of cell per gastric unit from ≥15 gastric units per mouse. D) Representative immunofluorescent staining of GIF (red color) and GS II (green color) on *H. pylori*‐infected *Tert^+/+^* and *Tert^−/−^* mice. Scale bar, 100 µm. E) Representative immunofluorescent staining of CD44v9 (red color) and H^+^K^+^ATPase (green color) on *H. pylori*‐infected *Tert^+/+^* and *Tert^−/−^* mice. Scale bar, 100 µm. F) Quantification of cell numbers per gland unit from panels D and E. Each group contains 5 mice. G) qRT‐PCR analysis for the expression of SPEM‐associated genes on *H. pylori*‐infected *Tert^+/+^* (*n* = 5) and *Tert* ^−/−^ mice (*n* = 5). *p*‐values: ^*^ *p* < .05; ^**^ *p* < .01; ^***^ *p* < .001, ns = not significant.

To further elucidate whether CagA‐induced SPEM was TERT‐dependent, we explored the effects of TERT depletion on the development of SPEM induced by *H. pylori^WT^* infection. To exclude the effect of *Tert* knockout on telomere length and mouse lifespan, all *Tert^−/−^* mice were derived from F1 generation mice crossed with heterozygous mice (*Tert^+/−^*). Immunofluorescence staining of gastric mucosa showed no substantial disparity in pit cell, parietal cell, SPEM cell, chief cell and neck cell numbers were observed between the baseline levels of wild‐type (*Tert^+/+^*) mice and *T* *ert* knockout (*Tert^−/−^*) mice (**Figure** **2**A–C). Compared with *H. pylori^WT^*‐infected *Tert^+/+^* mice, *H. pylori^WT^*‐infected *Tert^−/−^* mice showed significant relief from SPEM and parietal cell loss, including an increasing number of chief cells (Figure 2D–F). There was no notable variation observed in the number of neck cells between *Tert^+/+^* and *Tert^−/−^* mice infected with *H. pylori* *^WT^*. Consistent with this finding, the mRNA level of SPEM markers was downregulated in knockout mice and the expression of *Gif* was elevated (Figure 2G). These data indicated that CagA increased TERT protein abundance in chief cells and ameliorated SPEM via a TERT‐dependent manner.

**Figure 3:**
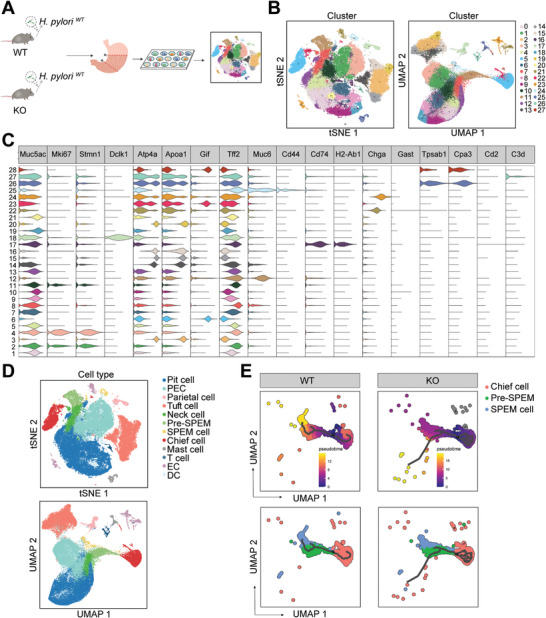
Cellular atlas of *H. pylori‐*infected *Tert* ^*+/+*^ and *Tert^−/−^* mice gastric glands. A) Schematic diagram of single‐cell RNA sequencing. Each group had 4 mice. B) t‐Stochastic neighbor embedding (t‐SNE) unbiased clustering and UMAP‐based unbiased clustering for the 76751 high‐quality cells from *H. pylori^WT^‐*infected *Tert^+/+^* (*n* = 3) and *Tert^−/−^* mice (*n* = 3). C) Violin plots of cell marker genes in 28 clusters. D) tSNE plots and UMAP plots showing cell types for the 76751 cells. E) Monocle3 trajectory UMAP plots based on differentially expressed genes in chief cells, pre‐SPEM cells and SPEM cells. PEC, proliferating epithelial cell; EC, enteroendocrine cell; DC, dendritic cell. WT, *Tert* *^+/+^*; KO, *Tert^−/−^*.

The single‐cell suspensions of corpus gastric mucosa from *H. pylori^WT^*‐infected *Tert* wild‐type mice and *Tert* knockout mice were obtained (**Figure** **3**A). After sequencing the transcripts of individual cells from each group, the data from each group was compiled and sent to an unbiased t‐SNE and UMAP dimension (Figure S3A,B, Supporting Information). The application of the clustering algorithm known as shared nearest neighbor resulted in the identification of 28 cell clusters across six samples (Figure 3B). The specific marker genes were used to identify the lineage of gastric epithelial cells, interstitial cells, and immune cells. Violin plots showed the expression of primary markers in 28 clusters (Figure 3C). The heat map further demonstrated the markers of chief cells, SPEM cells, parietal cells and neck cells (Figure S3C, Supporting Information). Cluster 6, 23 and 28 were chief cell because they only expressed highly chief cells’ markers. Cluster 25 was SPEM cell as it expressed both *Gif, Cd44* and *Muc6*. Furthermore, the expression of *Tff2* and other SPEM markers were highest in cluster 25. Between them, there was a population of transitional cells, cluster 12, which was defined as the pre‐SPEM cells. Finally, 12 types of cells were annotated (Figure 3D). Violin plots of primary markers’ expression showed their molecular signature well (Figure S4A, Supporting Information).

Pseudotime trajectory analysis of mucous chief cells, pre‐SPEM cells and SPEM cells elucidated the possible differentiation of SPEM during chronic *H. pylori* infection (Figure 3E). In *Tert* ^+/+^ mice, chief cells were identified as cellular origins of SPEM. While in *Tert* ^−/−^ mice, the differentiation trajectory of chief cell was disordered. This may be due to TERT‐regulated pathways were blocked after *Tert* knockdown and other signaling pathways are abnormally activated as compensation. And the branch of pre‐SPEM transdifferentiate into SPEM was blocked. This result suggested TERT had a substantial role in SPEM development.

### 2.4 Inhibition of the Wnt/β‐Catenin Pathway Leads to the Block of Chief Cell Transdifferentiation in *Tert* ^−/−^ Mice

**Figure 4:**
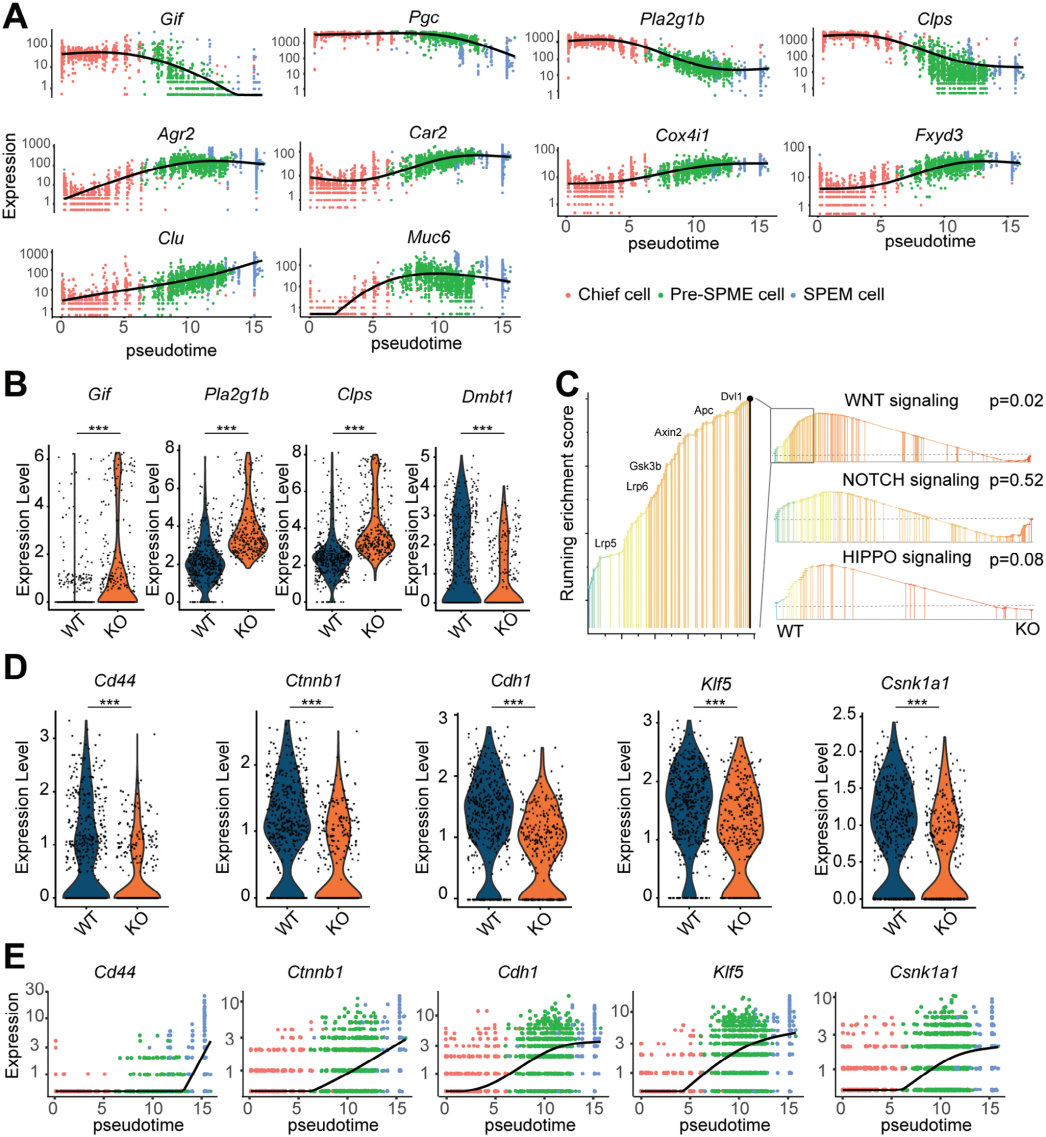
TERT prevents chief cells from dedifferentiating into SPEM cells by inhibiting Wnt signaling. A)Trend plots of top 10 differentially expressed genes in the pseudotime trajectory. B) Differential SPEM‐associated gene expression patterns of SPEM cells in *Tert* WT and *Tert* KO mice. C) Gene set enrichment analysis (GSEA) results showed the enrichment of Wnt pathway gene sets in SPEM cells of *Tert* KO mice. D) Violin plots for the expression of Wnt pathway‐associated transcripts *Cd44*, *Ctnnb1*, *Cdh1*, *Klf5*, and *Csnk1a1* in SPEM cells. E) Trend plots colored by gene expression for *Cd44*, *Ctnnb1*, *Cdh1*, *Klf5*, and *Csnk1a1*. *p*‐values: ^***^ *p* < .001.

To decode the molecular regulation mechanism in the formation of SPEM, differential expression genes drawing on a spatial correlation analysis across a single‐cell trajectory was tested in wild‐type mice. The top 10 differential genes of pseudoprime trajectory analysis further revealed the potential relationship of these three cells (chief cell, pre‐SPEM cell and SPEM cell) (**Figure** **4**A). Chief makers (*Gif*, *Pgc*, *Pla2g1b*, *Clps*, etc.) gradually decreased and metaplastic genes (eg, *Clu*, *Muc6*, etc.) gradually increased during the process of SPEM formation. The expression of *Agr2*, *Car2*, *Cox4i1* and *Fxyd3* were increased but their function in SPEM was not known. The newly identified genes included *Pgc*, *Pla2g1b, Clps*, *Agr2*, *Car2*, *Cox4i1*, and *Fxyd3*. The first three genes were upregulated in *Tert* ^−/−^ mice, while the remaining genes were downregulated upon qRT‐PCR analysis (Figure S4B, Supporting Information).These may be the key genes to the transdifferentiation of chief *cells* into SPEM cells. Similar changes were obtained with respect to the neck cell markers (*Tff2*, *Cela1*, *Gkn3*) or SPEM cell markers (*He4*, *Cftr, Cd44*) (Figure S4C–E, Supporting Information).

To further explore what effect the deletion of *Tert* on SPEM formation, the differential gene expression of SPEM cells between *Tert^+/+^* mice and *Tert^−/−^* mice was analyzed. Among the top 10 differential genes (Figure S4F, Supporting Information), several genes were associated with chief cells. Compared with wild‐type mice, SPEM cells retained more chief cells’ molecular characteristics in *Tert^−/−^* mice, like *Gif, Pla2g1b* and *Clps* (Figure 4B). While one of SPEM's markers, *Dmbt1* was decreased in *Tert^−/−^* mice. GSEA (Gene‐set enrichment analysis) demonstrated only Wnt/β‐Catenin pathway repressed in *Tert^−/−^* mice, without obvious affection of Hippo or Notch pathway (Figure 4C). qRT‐PCR results showed Wnt/β‐Catenin pathway‐related gene expression was reduced in *Tert^−/−^* mice (Figure S5A, Supporting Information). *Cd44*, *Ctnnb1*, *Cdh1*, *Klf5* and *Csnk1a1* were associated with Wnt/β‐Catenin.^[^ ^31^, ^32^, ^33^ ^]^ And their expression was decreased in *Tert^−/−^* mice (Figure 4D). Likewise, the transcriptional activity of these genes peaked in mice infected with *H. pylori^WT^* (Figure S5B, Supporting Information). Except they over‐expression in SPEM cells during the process of chief cells transdiffentiated into SPEM (Figure 4E). These results indicate that CagA‐increased TERT promotes the transformation of chief cells into SPEM cells by activating Wnt signaling pathway.

Additionally, the expression of inflammatory factors in *Tert^−/−^* mice was analyzed. Numerous cytokines were observed downregulated in *Tert^−/−^* mice (Figure S6A, Supporting Information). Notably, IL‐1 and IL‐17 were directly associated with the atrophy of parietal cells^[^ ^34^, ^35^ ^]^ and the IL‐1 and IL‐17 signaling pathways were affected by the *Tert* gene knockout. Validation by qRT‐PCR revealed *Tert* knockout significantly suppressed *Il1b* expression, without affecting *Il17a*, *Il17ra*, and *Il17rc* expression (Figure S6B, Supporting Information). This finding may account for the observed reduction in the number of apoptotic parietal cells in *Tert^−/−^* mice.

### 2.5 CagA Reduces TERT Ubiquitination by Inhibiting the Binding of SYVN1 to TERT

**Figure 5:**
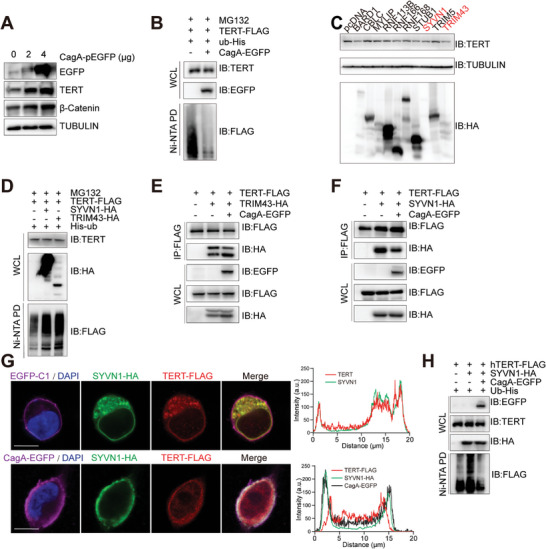
CagA reduces TERT ubiquitination by inhibiting the binding of SYVN1 to TERT. A) Western blot analyzed the transfection result of TERT and β‐Catenin in AGS cells at 48 h after transfection pEGFP‐CagA. B) Ubiquitination of TERT was tested 48 h after transfection with pEGFP‐CagA or pEGFP‐N1 in HEK293T cells in the presence of 10 µM MG132 for 8 h. C) Western blot detection of TERT in HEK293T cells after transfection with the indicated plasmids (*n* = 3). D) Ubiquitination of TERT was tested 48 h after transfection with SYVN1‐HA or TRIM43‐HA in HEK293T cells in the presence of 10 µmol L^−1^ MG132 for 8 h. E,F) Co‐IP analysis of the interaction between TERT‐Flag and TRIM43/SYVN1‐HA in HEK293T cells after transfection pEGFP‐CagA. G) Immunofluorescence analysis of TERT‐FLAG (anti‐FLAG, red color) and SYVN1‐HA (anti‐HA, green color) in HEK293T cells after transfection pEGFP‐CagA (Magenta color). Scale bar: 10 µm. H) Ubiquitination of TERT was tested 48 h after transfection with SYVN1 HA, SYVN1 Mut(C329S)‐HA, or the control in 293T cells in the presence of 10 µM MG132 for 8 h.

To further investigate the potential regulatory mechanism of CagA on TERT, AGS cell lines were transiently transfected with pEGFP‐CagA plasmids. CagA overexpression did not affect the mRNA levels of *TERT* (Figure S7A, Supporting Information), while remarkably facilitating TERT and β‐Catenin abundance (**Figure** **5**A). To examine the influence of TERT on the Wnt/β‐Catenin pathway, we performed a study where we assessed the TOP‐Flash reporter activity in AGS cells. TERT effectively hyperactivated the TOP‐Flash reporter in AGS cells treated with LiCl (Figure S7B, Supporting Information). These data suggested that TERT expression was regulated by CagA at the post‐transcriptional level. Moreover, a dramatic decreased ubiquitination level of TERT was observed in the presence of CagA, which indicated CagA‐inhibited TERT degradation was ubiquitination‐dependent (Figure 5B). Then we asked whether CagA downregulated the ubiquitination level of TERT by modulating the TERT‐E3 ligases binding. Nevertheless, no interaction between CagA and MDM2, MKRN1 and STUB1,^[^ ^36^, ^37^, ^38^ ^]^ which were previously validated as E3 ligases of TERT, was observed in co‐immunoprecipitation (Co‐IP) assays (Figure S7C, Supporting Information).

Therefore, an ON‐TARGET plus siRNA library containing 386 E3 ubiquitin ligases was applied to screen novel E3 ligases targeting TERT for degradation. SiRNA of 386 ubiquitin E3 ligase genes were individually transfected into 293T cells and endogenous TERT expression was assessed by Western blotting. Endogenous TERT was increased more than 1.5‐fold after the knockdown of 59 candidates (Figure S7D,E, Supporting Information and those with no obvious changes were not shown). Following previous methods,^[^ ^39^ ^]^ the ten candidates were further studied. SYVN1 and TRIM43 were selected as the most likely candidate E3 ligases (Figure 5C; Figure S8A, Supporting Information). Both of them could facilitate TERT ubiquitination (Figure 5D), while only SYVN1‐TERT interaction was inhibited in the presence of CagA (Figure 5E,F; Figure S8B, Supporting Information). TERT ubiquitination is reduced upon mutation of the enzyme's active site of SYVN1 (Figure S8C, Supporting Information). Similarly, the expression level and stability of endogenous TERT increased after SYVN1 knockdown by siRNA (Figure S8D,E, Supporting Information). Immunofluorescence showed the co‐localization of SYVN1 and TERT, but their co‐localization was reduced when CagA was expressed (Figure 5G). Meanwhile, CagA and SYVN1 were colocalized in the cytoplasm. Co‐IP also showed the binding of CagA and SYVN1 (Figure S8F, Supporting Information). Furthermore, the stabilizing effect of CagA on the TERT protein was lost upon SYVN1 knockdown (Figure S8G, Supporting Information). What's more, CagA reduced the ubiquitination of TERT by SYVN1 (Figure 5H; Figure S8H, Supporting Information). These results revealed CagA stabilized TERT proteins via disrupting the interaction between TERT and SYVN1.

### 2.6 NTZ Partially Reverses *H. Pylori*‐Induced SPEM via Inhibition of the Wnt/β‐Catenin Signaling Pathway In Vivo

Previously, it was reported that Nitazoxanide (NTZ), an antiparasitic medication authorized by the FDA, can hinder Wnt signaling by enhancing the citrullination of β‐Catenin and causing subsequent degradation.^[^ ^40^ ^]^ The following step involved examining whether the attenuation of SPEM in vivo was possible by inhibiting the Wnt signaling.

**Figure 6:**
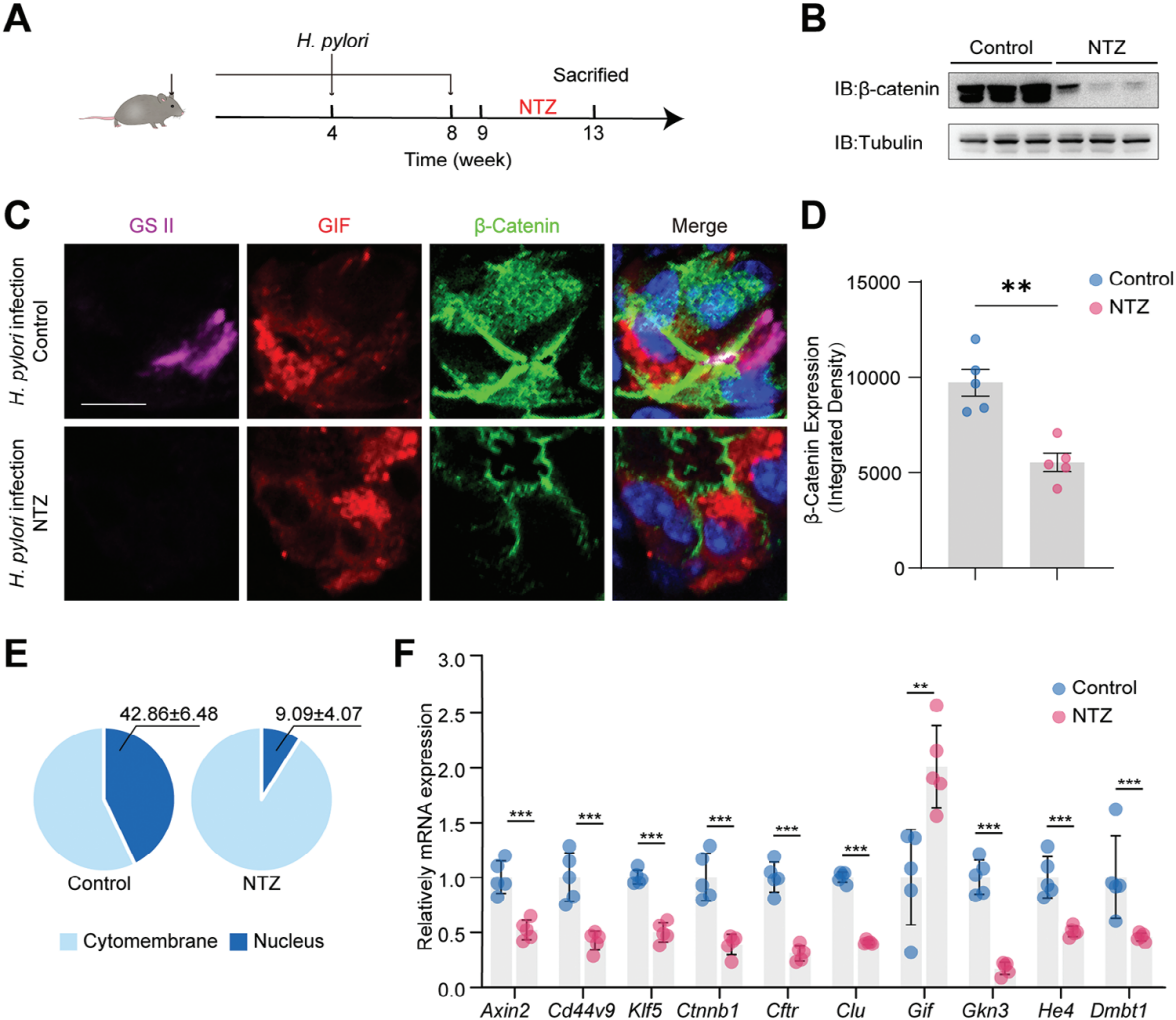
SPEM is resolved in *H. pylori*‐infected mice treated with nitazoxanide. A) Schematic diagram of the construction of *H. pylori*‐infected and nitazoxanide‐treated mouse models. Each group contains 5 mice. B) Western blot analysis of β‐Catenin protein in the gastric mucosa of the control and NTZ group. C) The immunofluorescent staining of GS II (magenta color), GIF (red color), and β‐Catenin (green color) in the control and NTZ group. Scale bar, 10 µm. D,E) Fluorescence integrated density and nuclear staining proportion of β‐Catenin from panel C. Each group had 5 mice. F) qRT‐PCR analysis for the expression of SPEM‐associated genes and β‐Catenin targeted genes in the control and NTZ group (*n* = 5). *p*‐values: ^**^ *p* < .01; ^***^ *p* < .001, ns = not significant. NTZ: nitazoxanide.

At the ninth week of *H. pylori* infection, mice were randomly assigned to receive either NTZ or placebo treatment for a total of 4 weeks dosing (**Figure** **6**A). *H. pylori* colonization had no significant difference after NTZ treatment (Figure S9A, Supporting Information). An obvious downregulation of β‐Catenin expression in gastric mucosa was validated after NTZ treatment (Figure 6B). In the gland’ base, wild‐type mice possessed a significant quantity of SPEM cells that were co‐stained with GS II and GIF. Conversely, NTZ‐treated mice exhibited a reduced number of SPEM cells along with a decrease in β‐Catenin expression (Figure 6C–E). Additionally, one month after treatment with NTZ in *H. pylori^WT^*‐infected mice, parietal cells, chief cells and neck cells were restored compared to the untreated group (Figure S9B–D, Supporting Information). The transcript levels of SPEM markers and β‐Catenin target genes (*Axin2, Cd44v9, Klf5, Ctnnb1*, *Cftr, Clu, Gkn3, He4* and *Dmbt1*) were lessened in NTZ group (Figure 6F). Collectively, these results demonstrate that NTZ effectively relieves SPEM signature via inhibition of the Wnt/β‐Catenin signaling pathway in vivo.

### 2.7 NTZ Reduces CagA‐Induced SPEM in Human Gastric Organoid

**Figure 7:**
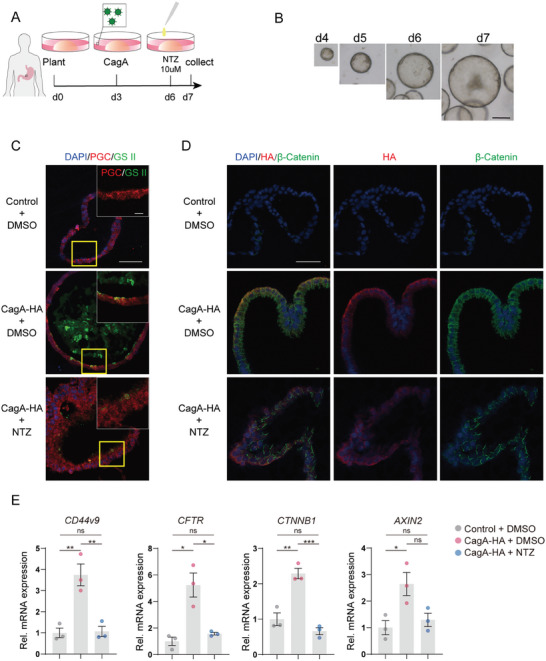
NTZ reduces CagA‐induced SPEM in human gastric organoid. A) Schematic diagram of the human gastric organoid culture. B) Representative light microscopic view of organoid growth. Scale bar, 100 µM. C) Representative immunofluorescent staining of GIF (red immunofluorescence) and GS II (green immunofluorescence) in the control, CagA‐HA lentivirus infection group and NTZ treatment group. Scale bar, 50 µm. D) Representative immunofluorescent staining of HA (red immunofluorescence) and β‐Catenin (green immunofluorescence) in the control, CagA‐HA lentivirus infection group and NTZ treatment group. Scale bar, 50 µm. E) qRT‐PCR analysis for the expression of SPEM‐associated genes and β‐Catenin targeted genes in the control, CagA‐HA lentivirus infection group and NTZ treatment group (*n* = 3). *p*‐values: ^**^ *p* < .01; ^***^ *p* < .001, ns = not significant. NTZ: nitazoxanide.

To explore the potential value of NTZ in clinical applications, organoids derived from human IM tissue were cultured for further investigation. Organoids were successively infected with CagA‐HA lentivirus on day 3 and treated with 10 µM of NTZ on day 6 for 24 h before being collected (**Figure** **7**A,B). In the organoid culture medium, the addition of Wnt3a protein resulted in the differentiation of most organoids into chief cells instead of pit cells, as demonstrated by immunohistochemistry (Figure S10A, Supporting Information). This is consistent with previous report.^[^ ^41^ ^]^ Under the light microscope, there were no noticeable differences in the growth rate and morphology of the three groups of organoids (Figure S10B, Supporting Information). Immunofluorescence staining showed that GS II protein was abundantly expressed in the organoids infected with CagA‐HA lentivirus, but not in the control group, meanwhile, some of the cells in the infection group were transformed from chief cells to SPEM cells. However, GS II expression was significantly reduced in organoids treated with NTZ (Figure 7C). Moreover, the β‐Catenin protein expression exhibited a notable rise in cells that were infected with CagA‐HA lentivirus but experienced a partial decline upon treatment with NTZ in organoids (Figure 7D). Moreover, qRT‐PCR also demonstrated that CagA promoted the expression of *CD44v9*, *CFTR*, *CTNNB1* and *AXIN2* in organoids while NTZ reversed this change to some extent (Figure 7E). These results comprehensively demonstrated that CagA promoted SPEM while NTZ inhibited this process by inhibiting Wnt signaling in human gastric organoids.

## 3 Discussion

In our study, *cagA*‐positive *H. pylori* infection successfully induced the occurrence of SPEM in the mouse gastric mucosa, characterized by the loss of parietal cells and chief cells, as well as the appearance of SPEM cells. *Tert*‐knockout mice and nitazoxanide treatment exhibited remarkable resistance against parietal cell loss and SPEM emergence in *H. pylori*‐infected mice. We demonstrate that CagA entering host cells initiates the novel mechanism of chief cells’ dedifferentiation. TERT plays an essential target for CagA, which inhibits the interaction of TERT and its E3 ubiquitin ligases, SYVN1. The gradual accumulation of TERT protein as a cofactor of β‐Catenin transcriptional complex upon nucleation promoted Wnt/β‐Catenin signaling. Metaplasia‐associated genes such as *Cd44*, *Ctnnb1* and *Sox9* were upregulated in chief cells and SPEM cells following activation of Wnt/β‐Catenin signaling. Remarkably, an FDA‐approved antiparasitic agent, Nitazoxanide (NTZ), dramatically ameliorated *cagA*‐positive *H. pylori*‐induced SPEM. In our research, we discovered a new way in which CagA enhances the Wnt signaling pathway (Graphical summary).

In addition to the classical function concerning the maintenance of telomere length, TERT is expressed primarily in germ cells, stem cells or progenitor cells to influence other biological behaviors. In the intestinal tract, TERT‐positive cells, stimulated by ROS‐HIFs‐transactivated Wnt2b signaling axis, switched from a quiescent state to a progenitor cell state that promotes intestinal regeneration after irradiation injury.^[^ ^42^ ^]^ The introduction of transgenic TERT expression in the kidney led to a notable increase in Wnt signaling and disruption of glomerular structure in mice. This indicates that TERT has a significant impact on the regulation of the Wnt signaling pathway in the kidney, ultimately affecting the structure and function of the glomeruli.^[^ ^43^ ^]^ Silencing TERT or suppressing Wnt signaling could normalize podiatric cells after glomerular injury. It has been reported that TERT physically interacts with Brahma‐related gene 1 (BRG1) and plays a supporting role in the β‐Catenin complex, leading to up‐regulation of genes in the Wnt network.^[^ ^27^ ^]^ Earlier research has indicated that the Wnt signaling pathway target genes are upregulated in the early stages after the deletion of parietal cells, while genes negatively regulating the Wnt pathway are downregulated.^[^ ^44^, ^45^ ^]^ Our data suggested that TERT played a crucial role in chief cell transdifferentiation via regulating Wnt signaling. TERT was overexpressed in chief cells during *cagA*‐positive *H. pylori* infection. After the knockout of *Tert* in mice, most chief cells stagnated at the pre‐SPEM stage and a decrease in SPEM population.

We presented the molecular foundation for utilizing NTZ as a drug for SPEM, which has the potential to enhance the clinical treatment of gastric intestinal metaplasia. Gastric intestinal metaplasia, as an important precancerous lesion of gastric mucosa, is an independent risk factor for intestinal gastric cancer, as well as a common digestive system disease, with a global incidence of 44.3%.^[^ ^46^ ^]^ However, the key molecular targets and mechanisms of gastric intestinal metaplasia are still unclear. Due to the lack of clear drug targets, there is no clear drug to reverse its progression. So far, patients with gastric intestinal metaplasia are mainly given regular follow‐up or radical treatment of *H. pylori*, but the benefits are not obvious.^[^ ^47^, ^48^ ^]^ The adhesin SabA of *H. pylori* interacted with sialyl‐Lewis X, allowing it to deeply infiltrate SPEM glands.^[^ ^49^ ^]^ This characteristic causes the *H. pylori* to expand from the pyloric to the corpus after SPEM. Therefore, relieving SPEM helps suppress the expansion of *H. pylori* in the stomach. The development of SPEM and the growth of gastric tumors are inhibited by genetically removing *Cd44* or using sulfasalazine, which is an inhibitor of xCT‐dependent cysteine transport.^[^ ^50^, ^51^ ^]^ Chief cells must control mitochondrial activity in response to ROS using a PGC1α‐xCT‐GPX4 axis and failed ROS scavenging blocks SPEM and promotes cell death by ferroptosis.^[^ ^52^ ^]^ An additional research group discovered that the MEK inhibitor Selumetinib is also efficacious in the treatment of SPEM. In gerbils infected with *H. pylori*, treatment with Selumetinib led to a reduction in SPEM and a restoration of oxyntic glands within the corpus mucosa, accompanied by a disappearance of phospho‐ERK1/2 immunoreactivity at the glandular base.^[^ ^53^ ^]^ NTZ was recently found to degrade β‐Catenin as an APC‐independent Wnt pathway inhibitor.^[^ ^40^ ^]^ Our research has found that nitazoxanide can act as an inhibitor of the Wnt pathway, alleviating the occurrence of SPEM. However, before applying NTZ in a clinical setting, several important factors should be taken into consideration. These include the stage of gastric intestinal metaplasia, potential long‐term side effects, and the existing health conditions of the patients. By carefully assessing these factors, healthcare professionals can determine the appropriateness and potential risks associated with the use of NTZ in each case.

Parietal cells play a pivotal role in the development of SPEM. Our findings indicate that TERT expression was absent in parietal cells, being predominantly localized to chief cells in both human and murine gastric tissues (Figure 1). Therefore, *Tert‐*knockout mice demonstrated a significant resistance against parietal cell loss and the onset of SPEM in *H. pylori*‐infected mice (Figure 2). In light of this observation, we postulated that there exists a form of cell‐cell communication among parietal cells, chief cells and SPEM cells. Many inflammatory factors, such as IL‐1, IL‐17, IL‐11, etc., can result in the atrophy of parietal cells. IL‐17A binds to IL‐17RA on parietal cells, activating caspase‐dependent apoptotic pathways that lead to the apoptosis of parietal cells.^[^ ^34^ ^]^ IL‐1β can act through IL‐1R1 on parietal cells to inhibit the expression of the Shh (Sonic Hedgehog) gene in parietal cells, leading to the atrophy of parietal cells.^[^ ^35^ ^]^ IL‐11 blocks gastric acid secretion by reducing the expression of parietal cell ion transport genes, CCKb, and histamine H2 receptors, inducing parietal cell loss.^[^ ^54^ ^]^ In our study, *Tert* knockout significant downregulation of IL‐1 pathway, without affecting IL‐17 and IL‐11 pathways. Therefore, further investigation is needed to identify why *Tert*‐knockout and NTZ protected oxyntic atrophy. A previous report suggested that the occurrence and progression of SPEM are not solely dependent upon the depletion of parietal cells.^[^ ^55^ ^]^ Metaplasia induction and macrophage polarization after parietal cell loss is coordinated through a cytokine signaling network of IL‐33 and IL‐13.^[^ ^56^ ^]^ Base cells within the corpus glands upregulated WFDC2/HE4 in response to gastric injury, which promotes SPEM by enhancing IL‐33 expression.^[^ ^57^ ^]^ Hence, we considered that the suppressive effects of TERT on SPEM partially rely on the transdifferentiation process of chief cells, and TERT indirectly modulated the fate of parietal cells by regulating inflammatory signaling pathways.

In summary, this article provides new insights into the clinical treatment of SPEM caused by *H. pylori* infection. We have discovered the molecular mechanism by which CagA promotes SPEM through facilitating the Wnt signaling pathway and demonstrated that NTZ could relieve SPEM in vivo.

## 4 Experimental Section

### Volunteers and Specimens

The study protocol received approval from Medical Ethics Committee of the Second Affiliated Hospital of Army Medical University (Chongqing, China). At Xinqiao Hospital, gastric biopsy samples and blood were obtained from 115 IM and 105 CG participants who underwent upper gastroduodenoscopy due to dyspeptic symptoms. The clinical information, entry criteria and exclusion criteria of patients were provided in Table S1 (Supporting Information). Two pieces of gastric mucosa from gastric body or angular incisure were obtained by biopsy clamps in intestinal metaplasia area and corresponding normal mucosa by endoscopy. One piece was processed for qRT‐PCR, and another was for immunohistochemistry (IHC) /immunofluorescence (IF) staining.

### *H. Pylori*‐Infected Mice Models and NTZ Treatment

C57BL/6J mice, both wild‐type (*Tert^+/+^*) and Tert knockout (*Tert^−/−^*), ≈8 weeks of age, were acquired (Suzhou, China). All *Tert* ^−/−^ mice were derived from F1 generation mice crossed with heterozygous mice (*Tert^+/−^*). In addition, heterozygotes were bred from wild‐type (*Tert^+/+^*) and heterozygotes. The mice were all maintained under a SPF environment under a 12 h light cycle. The mice fasted overnight and orogastrically inoculated 4 times every 12 h with 3 × 10^8^ CFU *H. pylori*.^[^ ^58^ ^]^ Mice were gavaged once a month for a total of three times. In NTZ treatment group, nitazoxanide (Aladdin, #N159057) (150 mg kg day^−1^) was administered by oral gavage for 4 weeks at the ninth week after infection with *H. pylori*. Mice were sacrificed painlessly in the thirteenth week.^[^ ^59^ ^]^

### *H. Pylori* Infection and *cagA* Gene Detection in Human

In clinical trials, *H. pylori* detection included rapid urease test, 13C‐UBT, Warthin‐Starry silver stain and *H. pylori*‐associated antibodies test. Using *H. pylori* antibody detection kit to test blood *H. pylori* CagA, VacA, UreB and UreA gene antibodies. A drop of fingertip blood was dropped into 1 mL of washing solution in *H. pylori* antibody detection kit (BLOT, #202201004) for the detection of *H. pylori‐*related antibodies (CagA, VacA, UreB and UreA antibodies) in the blood. Four diagnostic tests (rapid urease test, histology results, serum *H. pylori*‐associated antibodies test and C13 urea breath testing (UBT)) were used to determine whether patients were infected with *H. pylori*.

### *H. Pylori* Culture

*CagA*‐positive *H. pylori* PMSS1 (*H. pylori^WT^*) and *cagA*‐knockout *H. pylori* PMSS1 (*H. pylori* ^ΔcagA^) were grown in Skirrow blood plates under 37 °C microaerophilic conditions.^[^ ^60^ ^]^ The colonies are colorless, transparent and pinpointed in size. Replace the blood plate at least 48 h to prevent *H. pylori* ball change. After gram staining, *H. pylori* showed red and had 2–3 curves under oil microscope.

### *H. Pylori* Colonization Detection in Mice

Mouse's gastric antrum tissues were collected, then cut into small pieces. The genomic DNA was obtained using a kit specifically designed for genomic DNA extraction (Tiangen, #DP304). According to previous method,^[^ ^61^ ^]^ the colonization of *H. pylori* could be measured by TaqMan method using primers and probe of *H. pylori*‐specific 16S rDNA (Table S2, Supporting Information). Standardizing the data using the previous method.^[^ ^58^, ^62^ ^]^

### Cell Culture and Transfection

The HEK293T cells (ATCC, #CRL‐3216) and AGS cells (ATCC, #CRL‐1739) were cultured in Dulbecco's Modified Eagle Medium (DMEM) or DMEM/F‐12 (Nutrient Mixture F‐12) supplemented with 10% fetal bovine serum and 1% penicillin‐streptomycin. The cells were kept at a temperature of 37 °C in a moist environment with 5% carbon dioxide. Cells were left to adhere for at least 18 h before transfection. The plasmid, serum‐free medium, and Lipofectamine 8000 Reagent (Beyotime, #C0533) were mixed evenly, and then evenly dripped into the culture dish. Fresh medium was replaced 4–6 h later. RNA was extracted at 24–36 h and protein was extracted at 48–72 h.

### Immunofluorescence Staining

Mouse stomach was incised along the greater curvature, with the antrum and the forestomach removed. Sections of Mouse Corpus or human mucosa biopsies were preserved by immersing them in 4% paraformaldehyde at a temperature of 4 °C for a duration of 12 h. The preserved samples were then encased in wax/paraffin and divided into sections measuring 2.5 µm. The sections were dewaxed with xylene and rehydrated with gradient alcohol. Then antigen repair was performed by EDTA buffer (pH 9.0). After this, 5% goat serum was used to block for 30 min. Primary antibodies were diluted in PBS buffer and incubated overnight at 4 °C. The sections were washed with PBS three times, then secondary antibodies were applied for 1 h at 37 °C. Detail Information on primary antibodies and secondary antibodies was provided in the Table S3 (Supporting Information).

### Immunofluorescence Quantification and Quality Control

All quantifications encompassed at least five samples. Quantifications were done on patients’ gastric samples stained fluorescently for anti‐H^+^/K^+^ATPase, GS II lectin, and anti‐PGC. H^+^/K^+^ATPase positive cells were considered as parietal cells, PGC positive cells as chief cells, GS II positive cells as neck cells, and PGC^+^/GS II^+^ double‐positive cells considered transitional or SPEM cells. In mice, the chief cells were marked with GIF.^[^ ^55^, ^63^ ^]^ Previous research has indicated that mature parietal cells, in contrast to their counterparts at other developmental stages, were distinguished by several morphological and functional enhancements. These cells exhibit a significant increase in cellular volume, a centripetal migration of the nucleus towards the cell center, and an elevated mitochondrial content.^[^ ^64^ ^)^ Notably, the dimensions of mature parietal cells were considerably more substantial, with the greatest diameter surpassing 14 µm.^[^ ^65^ ^]^ Therefore, only the parietal cells with a diameter greater than 14 µm were counted.

### Immunohistochemistry Staining

The sections were placed in a solution of 3% hydrogen peroxide and incubated for 10 min at room temperature. The primary antibodies were diluted in TBS solution and left to incubate at 4 °C overnight. Second antibodies were then applied the next day and incubated at 37 °C for 30 min. The AEC (3‐amino‐9‐ethyl carbazole) (Solarbio, #A2010) or DAB (3, 3‐diaminobenzidine) (Beyotime, #P0202) was then added and reacted with HRP (horse radish peroxidase) for 5–15 min. Afterwards, the sections were stained with hematoxylin for a duration of 1 min, followed by differentiation using hydrochloric alcohol for 5 s, and subsequently washed with water for a period of 5 min. Finally, the sections were sealed with neutral resin containing xylene/glycerin jelly.

### RNA Extraction and qRT–PCR

For total RNA isolation from cells/tissue, RNAiso Plus reagent (Takara, #9109) was employed. The extracted RNA was then reverse transcribed into cDNA using the PrimeScript RT Reagent Kit (Takara, #RR047A). The resulting cDNAs were subjected to quantitative real‐time PCR (qRT‐PCR) using SYBR Premix Ex Taq II (Takara, #RR820A). The primer sequences were provided in Table S2 (Supporting Information).

### Western Blotting

Proteins were extracted using RIPA buffer supplemented with a protease inhibitor. Protein concentrations were measured by employing an improved BCA protein assay kit (Beyotime, #P0009). The Western blot analysis was conducted following the previously described method.^[^ ^39^ ^]^ Antibodies’ information was shown in Table S3 (Supporting Information).

### ScRNA‐Sequencing Samples Preparation

The gastric body tissue was washed gently with ice‐cold PBS (phosphate buffered saline) until the solution contained no impurities. The oxyntic mucosa glands were harvested with a cell scraper to separate the mucosa and serosa. Then glands were collected and digested in 2 mL of Tissue Dissociation Solution (Singleron Biotechnologies, SCelLiVe Debris Removal Kit) in a shaker at 37 °C, 100 rpm min^−1^ for 15 min. It was stained 10 µL suspension with Trypan Blue and weighed them in a counting chamber under the microscope to assess cell activity and density. After filtering through a 70 µm sterile strainer, red blood cell lysis buffer was added to remove red blood cells. Finally, cells were resuspended in PBS at 1 × 10^5^ cells mL^−1^.

### ScRNA‐Sequencing and Quality Control

The single‐cell suspension was then loaded onto the microfluidic chip and scRNA‐seq libraries were constructed using the GEXSCOPE Single‐Cell RNA Library Kit (Singleron Biotechnologies, #5180021) according to the Singleron GEXSCOPE protocol. The resulting scRNA‐seq libraries were sequenced on an Illumina HiSeq X10 instrument with 150 bp paired end reads. The raw reads underwent processing with fastQC and fastp to eliminate readings of poor quality. Before aligning read two to GRCm38 using Ensemble v.92 gene annotation, the trimming of adapters and poly A tails was performed using fastp V1 (version 2.5.3a and feature Counts 1.6.2). Groups were formed by matching the cell barcode, UMI, and gene to determine the count of UMIs for each gene in each cell. The count table of each cellular barcode's UMI was saved for future analysis.

The cells that had expressed genes below 200 or above 7500 was removed. Additionally, any cells containing over 25% of mitochondrial genes were excluded. Cells with more than 25% mitochondrial genes were also removed. After quality control, a total of 76751 cells were obtained for the subsequent analysis. Data were stored in NGDC database (PRJCA016527, https://ngdc.cncb.ac.cn/). Chief cells were marked by *Cryap*, *Necab1*, *Clps*, *Pla2g1b*, *Gif* and *Pniliprp2*. Neck cells were marked by *Tff2*, *Muc6*, *Cela1 or Gkn3*.^[^ ^66^ ^]^

### SiRNA Library Screening for E3 Ligases Targeting TERT Protein

HEK293T cells were plated in 96‐well dishes with a cell density of 4 × 10^3^ cells per well and cultured until reaching 50% confluency. Then the ON‐TARGET plus siRNA library (Dharmacon, #5180021) containing 386 E3‐targeting siRNAs was employed as previously described. To summarize, the lipofectamine 3000 transfection reagent from Invitrogen (Invitrogen, #L3000008) was thinned down in Opti‐MEM and introduced into every well together with the siRNA pool. After incubating for 15 min, the mixture for transfection was moved to HEK293T cells, and all siRNAs were transfected with a concentration of 50 nM. After another 48 h, the cells were collected for endogenous TERT expression detection via western blot assay. The novel E3 ligases specific screening procedure was performed in a previous study.^[^ ^39^ ^]^ The quantification of band intensities was performed with ImageJ software to select E3 ligase candidates which decrease TERT abundance more than 1.5‐fold. Afterwards, the E3 ligase prediction tools UbiBrowser (http://ubibrowser.ncpsb.org.cn/ubibrowser/) and UbiNet 2.0 (http://awi.cuhk.edu.cn/~ubinet/index.php) were used to indicated E3 ligase candidates potentially degrade TERT.

### Co‐Immunoprecipitations (Co‐IP)

The Co‐immunoprecipitation (Co‐IP) tests were conducted according to the method described beforehand.^[^ ^67^ ^]^ Following the addition of EBC buffer (composed of 50 mM Tris pH 7.5, 120 mM NaCl, and 0.5% NP‐40) along with protease inhibitors to the cellular precipitate, the samples were vigorously mixed and agitated for a duration of 30 s, subsequently undergoing an incubation period of 15 min at a temperature of 4 °C while being continuously rotated. After being spun at a speed of 13 000 revolutions per minute for a duration of 10 min, the resulting liquid lysates were gathered. To determine the levels of complete protein, one‐fifth of the lysate was utilized, while two‐fifths of the lysate were subjected to incubation with anti‐HA or anti‐Flag agarose beads at a temperature of 4 °C for a duration of 3 h. After incubation, the mixtures were centrifuged at 13 000 rpm min^−1^ for 30 s, then the supernatant was removed carefully. 1 mL NETN buffer (20 mM Tris, pH 8.0, 150 mM NaCl, 1 mM EDTA and 0.5% NP‐40) was added to wash the agarose beads. In the end, western blotting was used to detect proteins that were co‐immunoprecipitated.

### Ubiquitination Assays

The ubiquitination tests were executed according to the previously stated method.^[^ ^68^ ^]^ HEK293T cells were transfected with the His‐ubiquitin and other plasmids for a duration of 36 h. Prior to harvest, the cells were treated with 10 µmol of MG132 (Beyotime, #S1748) for 8 h. To collect the cells, Buffer A (comprised of 6 M guanidine‐HCl, 0.1 M Na_2_HPO_4_/NaH_2_PO_4_, and 10 mM imidazole [pH 8.0]) was used followed by sonication on ice. Next, the lysates were incubated with nickel‐nitrilotriacetic acid (Ni‐NTA) matrices (QIAGEN, #30210) at room temperature for 3 h. Afterwards, the lysates were subjected to consecutive rinsing with buffer A, buffer A/TI (with a ratio of 3 parts buffer A to 1 part buffer TI), and buffer TI (containing 25 mM Tris‐HCl and 20 mM imidazole [pH 6.8]). In the end, the proteins acquired from the pull‐down were separated using SDS‐PAGE and then analyzed through immunoblotting.

### Human Gastric Organoid Culture

The organoid culture was executed according to the previously stated method.^[^ ^41^, ^69^ ^]^ Two biopsies from the area of intestinal metaplasia were taken endoscopically and rinsed softly with ice‐cold PBS to clean the surface impurities. Tissues were digested with 10 mM EDTA solution for 40 min on a shaker at 4 °C. The tissue was then extruded to completely separate the gastric glands. The gastric glands were resuspended in matrigel (BD, # 356231) and then cultured in advanced DMEM/F12 (Gibco, #126340101) medium containing penicillin/streptomycin, 10 mmol L^−1^ HEPES, 200 mmol/L GlutaMAX, 1 × B27 (Gibco, #17504044), and 1 mmol L^−1^ N‐acetylcysteine (MCE, #HY‐B0215), 50 ng mL^−1^ epidermal growth factor (EGF) (bioGenous, #568), 100 ng mL^−1^ noggin (bioGenous, #807), 500 ng mL^−1^ R‐spondin1 (bioGenous, #861), 100 ng mL^−1^ Wnt‐3a (bioGenous, #5036), 200 ng mL^−1^ fibroblast growth factor (FGF)10 (bioGenous, #816), 1 nmol L^−1^ gastrin (Absin, #abs45129455), 2 mmol L^−1^ TGFβ inhibitor (MCE, #A‐83‐01) and 10 mmol L^−1^ rho‐associated coiled coil forming protein serine/threonine kinase (RHOK) (Sigma–Aldrich, #Y‐27632).

### Statistical Analysis

Statistical analyses were performed using GraphPad Prism (V9.0), R (V4.1), ImageJ (V1.52) and Image Pro Plus (V6.0). The statistical differences between the two groups were determined by Student's t‐test or Welch's t‐test. One‐way ANOVE was used for comparisons among multiple groups. The level of significance was indicated as ^*^ *p* < 0.05, ^**^ *p* < 0.01, ^***^ *p* < 0.001.

## Conflict of Interest

The authors declare no conflict of interest.

## Author Contributions

L.H., X.Z. and S.Z contributed equally to this work. The experimental design and manuscript revisions were carried out by S.Y., C.H., and Y.L. The order of co‐first authors L.H., X.Z., and S.Z. was determined based on their contributions to the article. L.H. conducted the majority of the experiments, wrote the majority of the manuscript text, and analyzed the data. S.Z. and X.Z. conducted some experiments and contributed to the manuscript writing. Y.W. performed the ScRNA‐sequencing analysis. W.H., J.L., Y.L., Y.L., X.P., J.L., H.Z., and L.W. participated in certain experiments. All authors read and approved the final version of the manuscript.

## Supporting information



Supporting Information

## Data Availability

The data that support the findings of this study are openly available in NGDC database at https://ngdc.cncb.ac.cn/, reference number 16527.
