# Supplementary material for: H. Pylori‐Facilitated TERT/Wnt/β‐Catenin Triggers Spasmolytic Polypeptide‐Expressing Metaplasia and Oxyntic Atrophy
Source: Adv Sci (Weinh). 2024 Nov 25;12(3):2401227. doi: 10.1002/advs.202401227 (PMC11744579; doi:10.1002/advs.202401227)

Supplementary Materials

***H. pylori*-facilitated TERT/Wnt/β-Catenin triggers Spasmolytic Polypeptide-Expressing Metaplasia and Oxyntic Atrophy**

*Lijiao He^*^, Xiao Zhang^*^, Shengwei Zhang^*^, Yi Wang, Weichao Hu, Jie Li, Yunyi Liu, Yu Liao, Xue Peng, Jianjun Li, Haiyan Zhao, Liting Wang, Yang-fan Lv^2^†, Chang-jiang Hu†, Shi-ming Yang†*

| **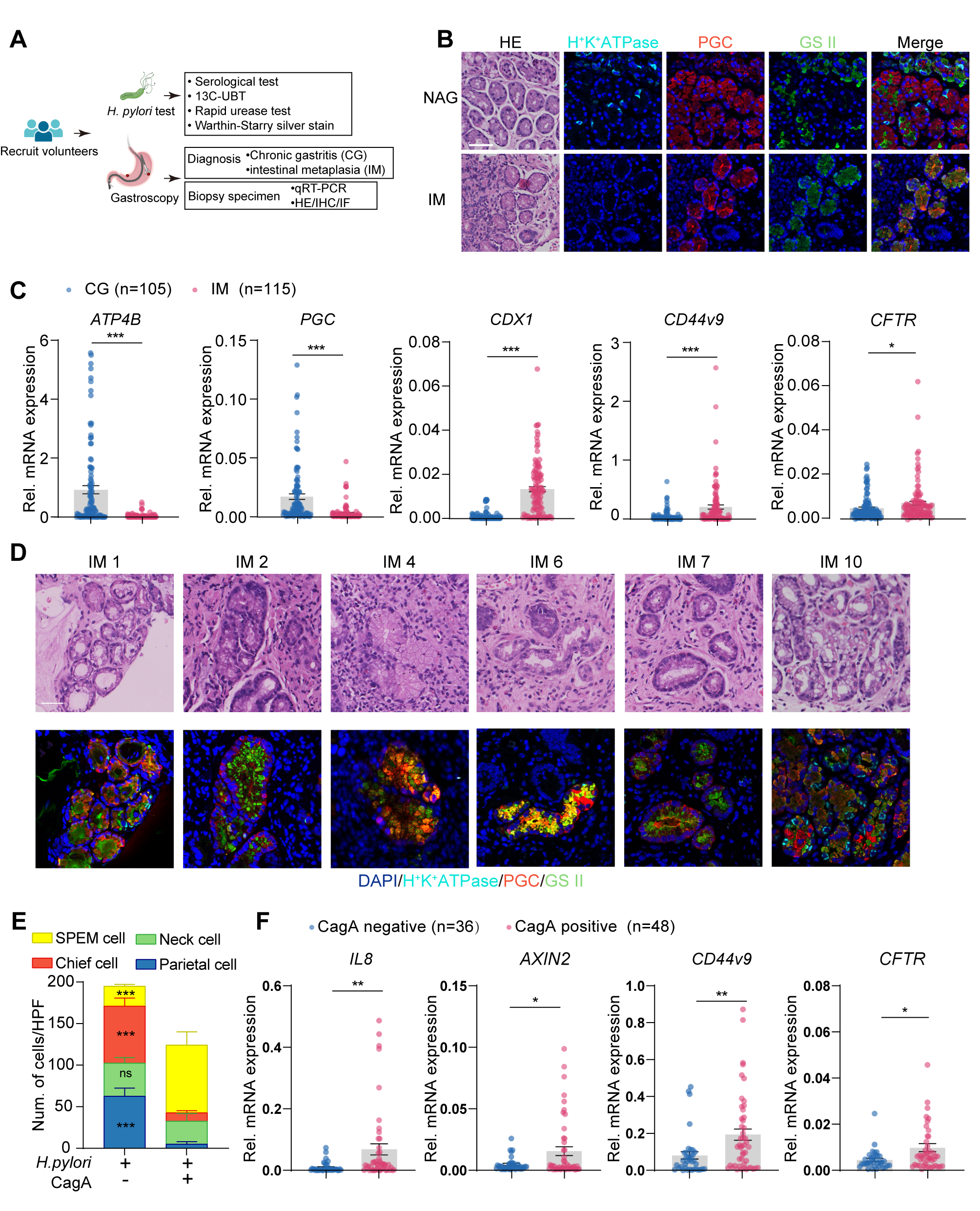** |
| --- |
| **Figure S1. Infection with CagA-positive *H. pylori* promotes SPEM in human intestinal metaplasia gastric mucosa. (A)** Flowchart for collection and tests of human samples. **(B)** Representative HE staining and immunofluorescence staining of human gastric oxyntic mucosa. The immunofluorescence staining of DAPI (blue color), H^+^K^+^ATPase (cyan color), PGC (red color) and GS-II (green color) in the CG and IM groups, respectively. Scale bar, 50 µm. **(C)** qRT-PCR analysis for the expression of genes related to intestinal metaplasia and SPEM on human gastric oxyntic mucosa. The CG group contained 105 patients and the IM group contained 115 patients. **(D)** Representative patient HE staining and immunofluorescence staining of PGC (red color), GS-II (green color) and H^+^K^+^ATPase (cyan color) in IM samples. Scale bar, 50 µm. **(E)** The cell numbers of immunofluorescent staining of panel D in the CagA-negative/positive *H. pylori-*infected IM patients. *: Significance between two groups. **(F)** qRT-PCR analysis for the expression of *IL-8*, *AXIN2*, *CD44v9* and *CFTR* on the *cagA*-negative *H. pylori*-infected (n=36) and *cagA*-positive *H. pylori*-infected (n=48) IM patients’ gastric oxyntic mucosa. *P*-values: **P* < .05; ***P* < .01; ****P* < .001, ns=not significant. CG, chronic gastritis; IM, intestinal metaplasia. Errors bars represent mean ± SEM of biological replicates. |

| 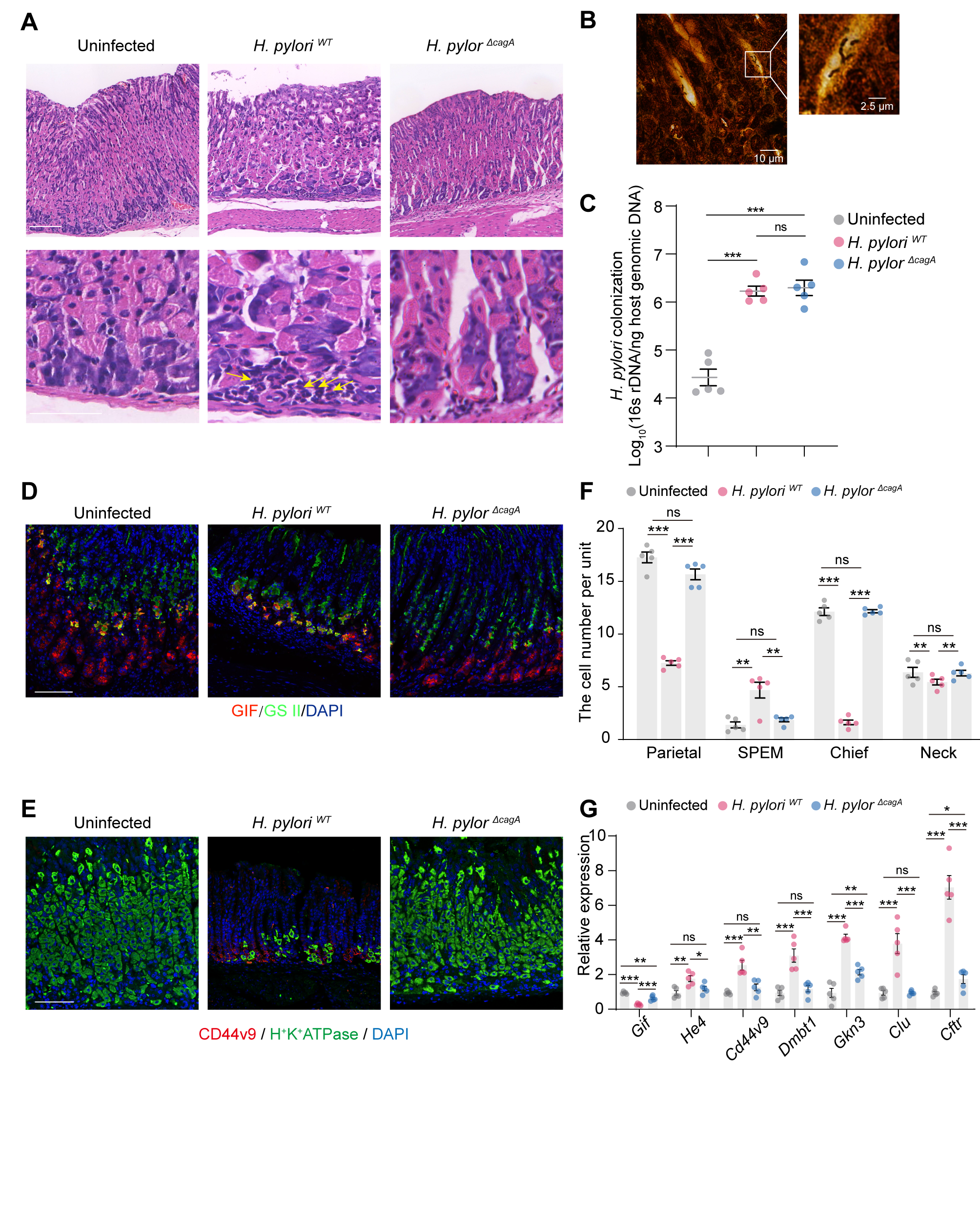 |
| --- |
| **Figure S2. CagA-positive *H. pylori* infection promotes SPEM in mice gastric mucosa.** **(A)** Representative HE staining of gastric mucosa on uninfected, *H. pylori^WT^* infected and *H. pylori^ΔcagA^* infected mice. The yellow arrows show immune cells. Scale bar, 50 µm. **(B)** Warthin-Starry silver staining of gastric mucosal tissue in *H. pylori*-infected mice. **(C)** Probe-PCR for the colonization amount of *H. pylori* in gastric mucosa on uninfected, *H. pylori^WT^*-infected and *H. pylori^ΔcagA^*-infected mice. **(D)** Representative immunofluorescent staining of GIF (red color) and GS-II (green color) on uninfected, *H. pylori^WT^* infected and *H. pylori*^ΔcagA^ infected mice. Scale bar, 100 µm. **(E)** Representative immunofluorescent staining of CD44v9 (red color) and H^+^K^+^ATPase (green color) on uninfected, *H. pylori^WT^* infected and *H. pylori^ΔcagA^* infected mice. Scale bar, 100 µm. **(F)** Quantification of cell numbers per gland unit from panels D and E. Each group contains 5 mice. Each data point represents the mean number of each type of cell per gastric unit from ≥15 gastric units per mouse. **(G)** qRT-PCR analysis for the expression of SPEM-associated genes on uninfected, *H. pylori^WT^* infected and *H. pylori*^ΔcagA^ infected mice (n=5). *P*-values: **P* < .05; ***P* < .01; ****P* < .001, ns=not significant. |
|  |

| **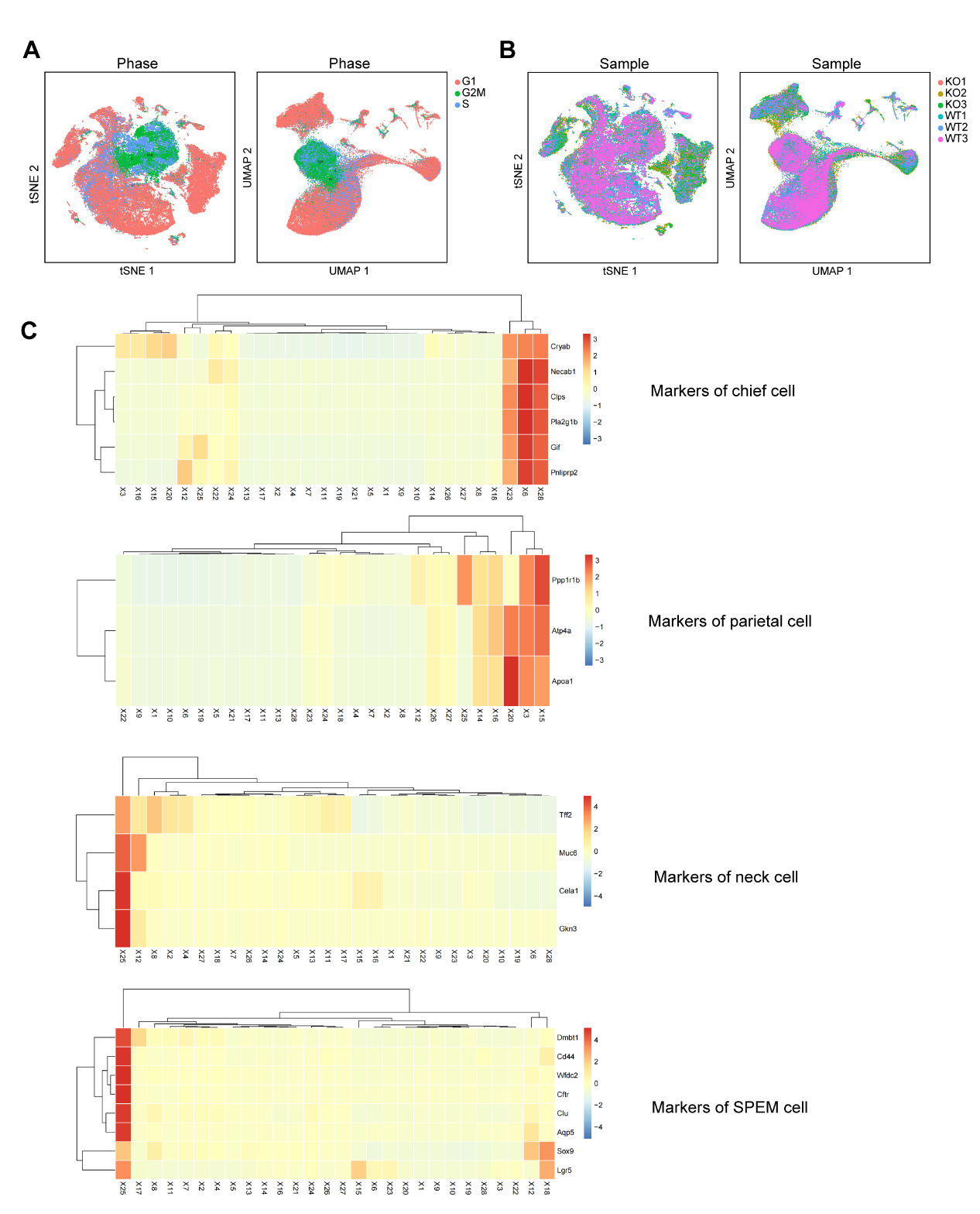** |
| --- |
| **Figure S3. Cellular atlas of scRNA-sequencing. (A-B)** t-Stochastic neighbor embedding (t-SNE) and UMAP-based unbiased clustering for the 76,751 high-quality cells showing cell cycle phase and sample origin. **(C)** Heatmap showing the expression of marker genes in four cell types. |

| **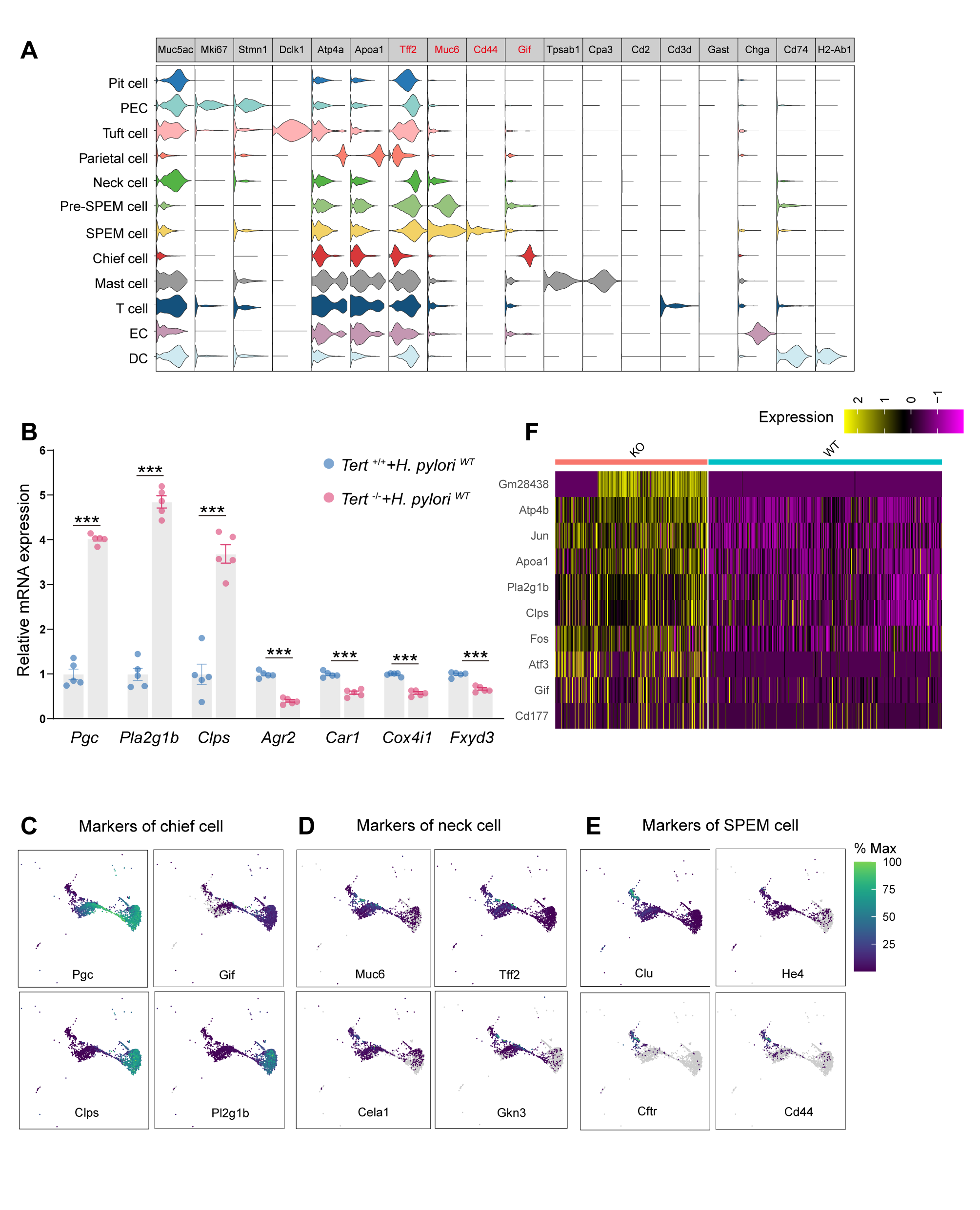** |
| --- |
| **Figure S4. Differential genes of SPEM cells between WT and KO mice. (A)** Violin plots showing the smoothed expression of cell marker genes in 12 cell types. **(B)** qRT-PCR analysis for the expression of differentially expressed genes in the pseudotime trajectory. **(C-E)** Feature trajectory UMAP plots of important markers in three cell types. **(F)** Top 10 differentially expressed genes of SPEM cells between KO and WT mice. |

| **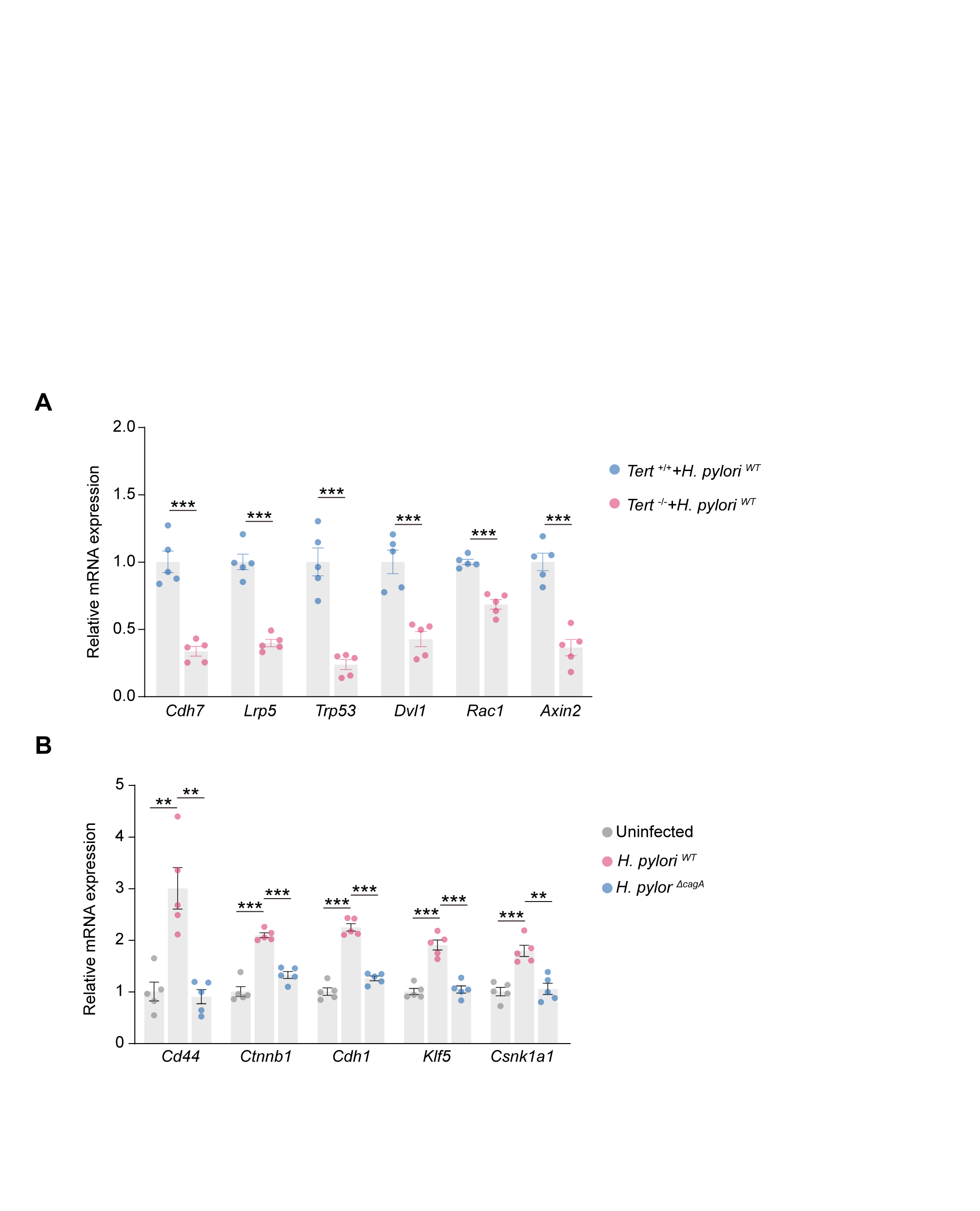** |
| --- |
| **Figure S5. Wnt/β-Catenin pathway-related genes expression.** **(A)** qRT-PCR analysis for the expression of Wnt/β-Catenin pathway-related genes on *H. pylori*-infected *Tert^+/+^* (n=5) and *Tert^-/-^* mice (n=5). **(B)** qRT-PCR analysis for the expression of Wnt/β-Catenin pathway target genes on uninfected (n=5), *H. pylori^WT^* infected (n=5) and *H. pylori*^ΔcagA^ infected (n=5) mice. *P*-values: ****P* < .001; ***P* < .01. |

| **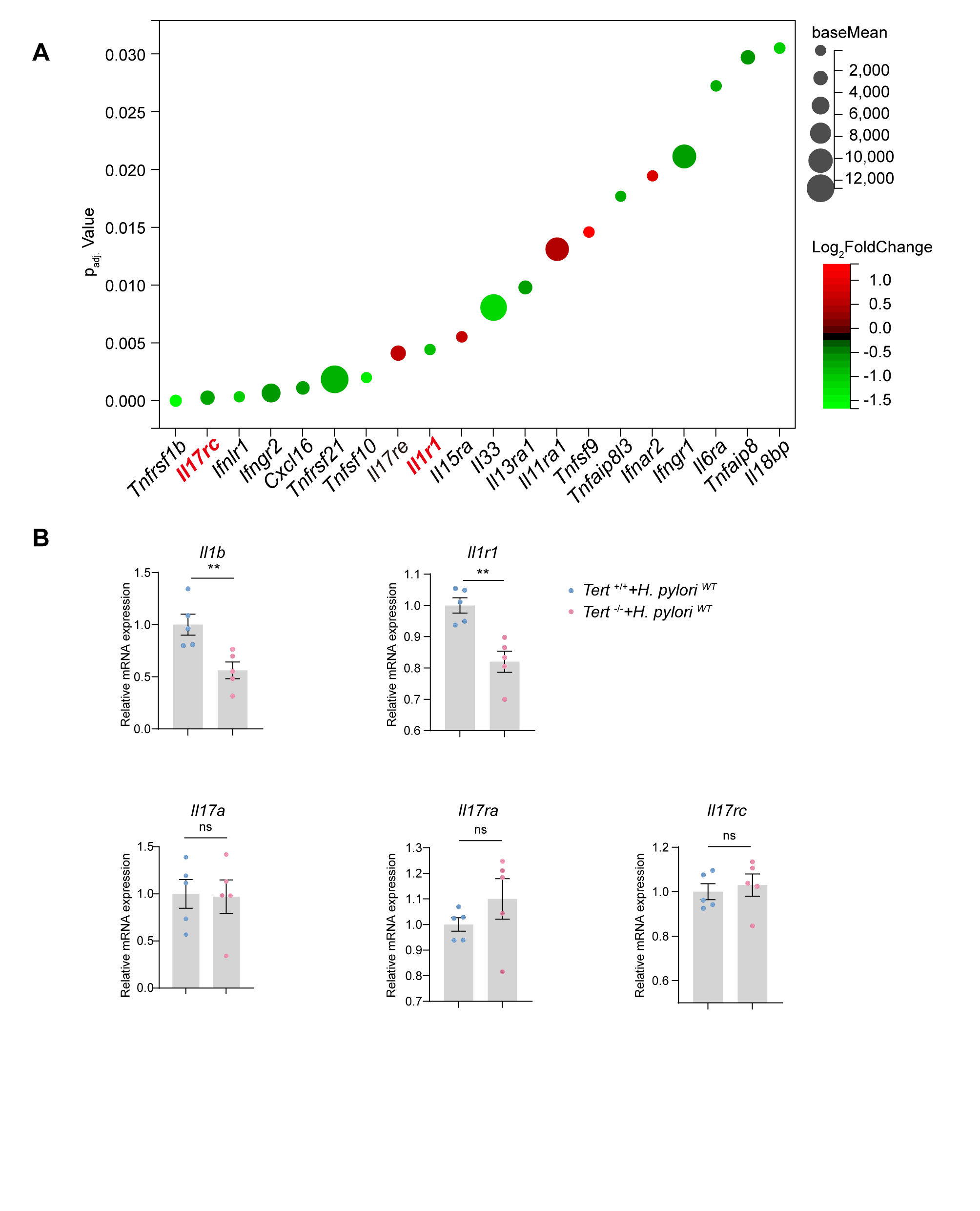** |
| --- |
| **Figure S6. Cytokine related genes expression.** **(A)** Top 20 differentially expressed cytokines-related genes between KO and WT mice in scRNA-sequencing using pseudobulks differential expression analysis. **(B)** qRT-PCR analysis for the expression of cytokine related genes on *H. pylori*-infected *Tert^+/+^* (n=5) and *Tert^-/-^* mice (n=5). *P*-values: ***P* < .01. |

| **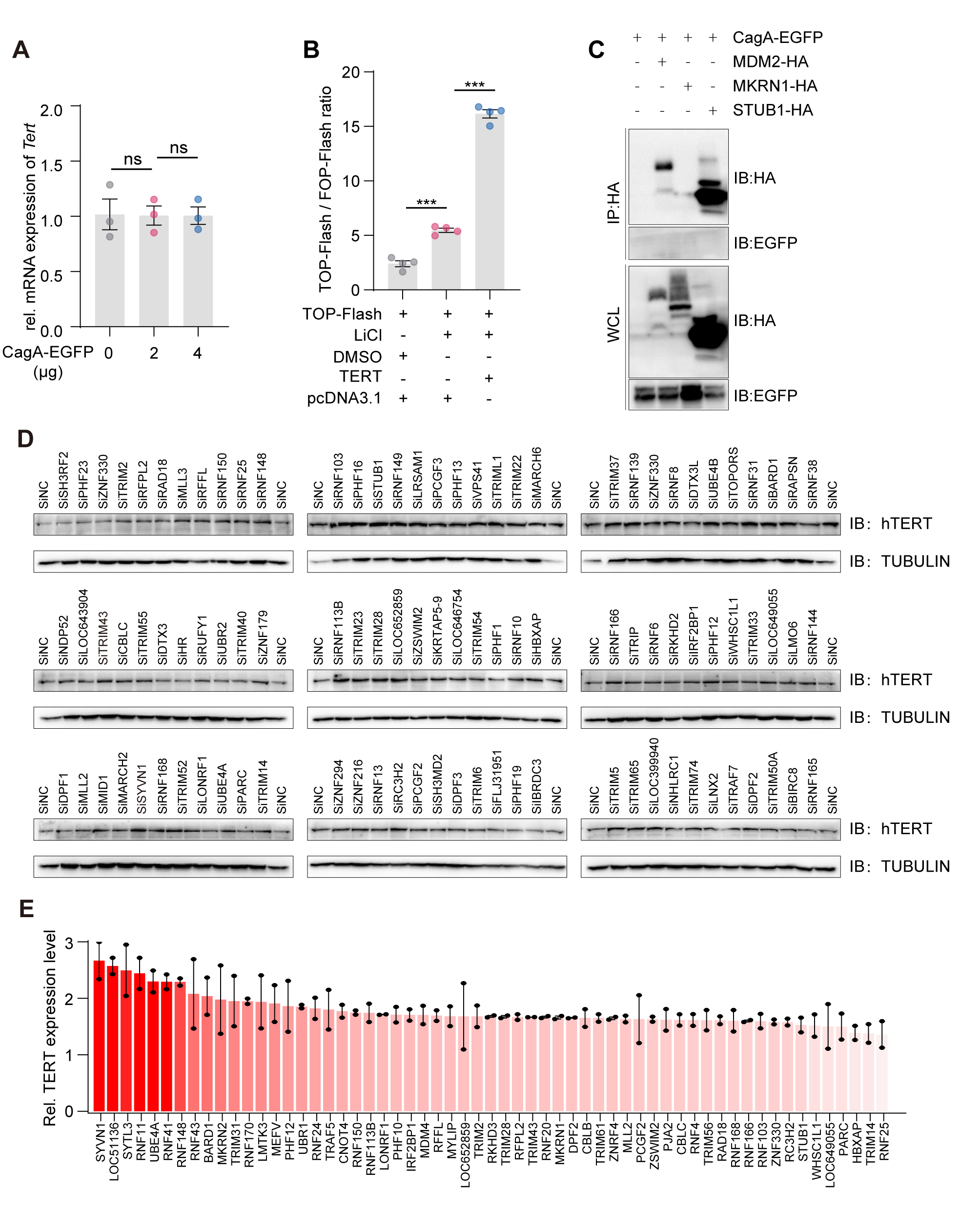** |
| --- |
| **Figure S7. Novel E3 ligase screening process of TERT. (A)** qRT-PCR analyzed the transfection results of TERT and β-Catenin in AGS cells at 48 hours after transfection with pEGFP-CagA (n=3). **(B)** TOP-Flash activity in AGS cells co-transfected with empty vector or human TERT expression plasmid and treated with or without LiCl (n = 4). **(C)** Co-IP analysis of the interaction between CagA-EGFP and E3-HA in HEK293T. **(D)** Western blot analyzed the siRNA transfection result of TERT in HEK293T cells after knockdown of indicated E3 ligase. **(E)** Relative TERT protein expression quantified by Image J software after the knockdown of indicated E3 ligase (n=2). |

| 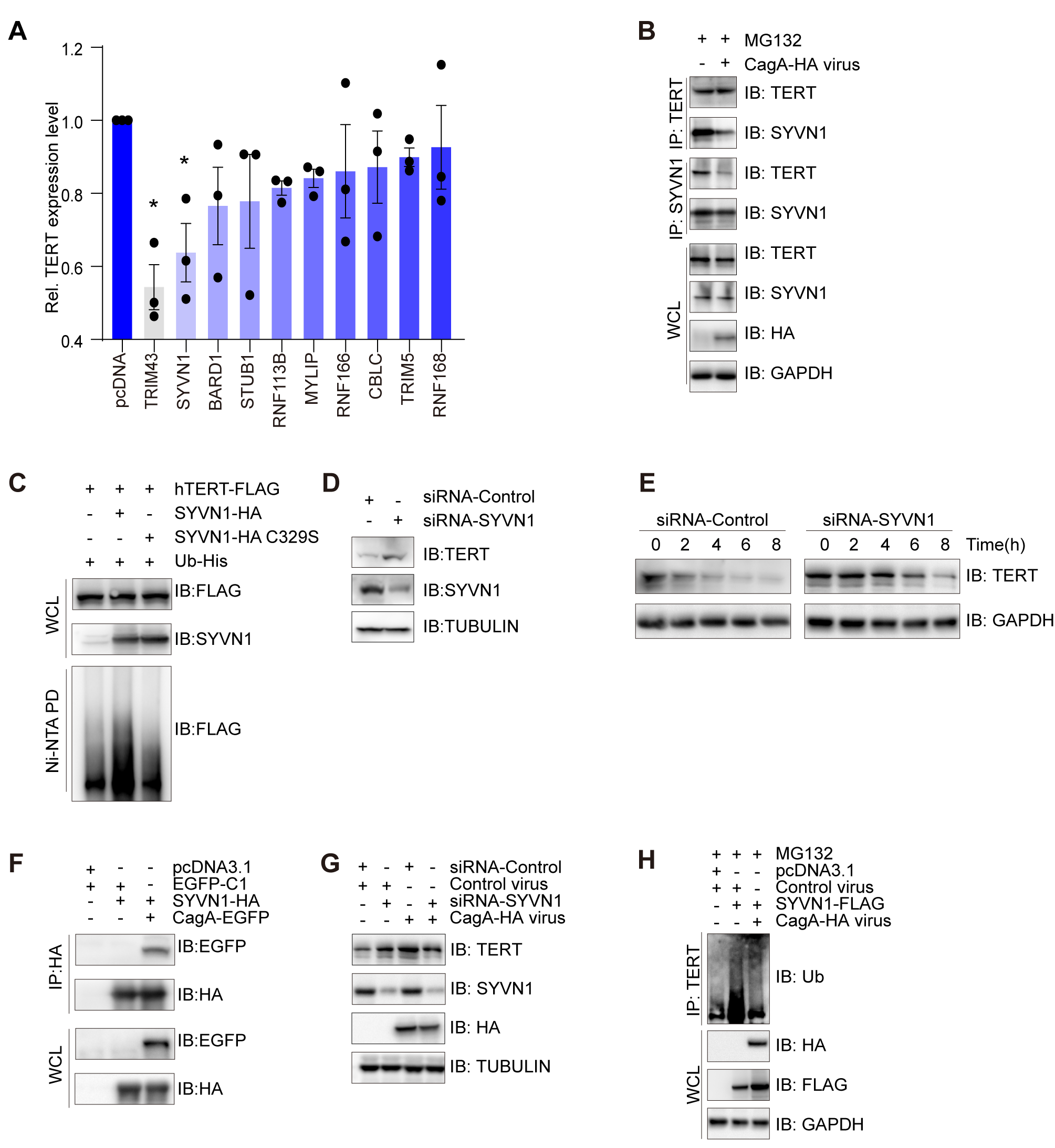 |
| --- |
| **Figure S8. SYVN1 is a novel E3 ligase of TERT.** **(A)**Western blot quantification of TERT in HEK293T cells after transfection with the indicated plasmids (n=3). **(B)** The co-immunoprecipitation experiments showed the impact of CagA on the endogenous binding of SYVN1 and TERT in AGS cells. **(C)** Ubiquitination of TERT was tested 48 h after transfection with SYVN1-HA, SYVN1 Mut(C329S)-HA, or the control in 293T cells in the presence of 10 μM MG132 for 8 h. **(D)** Western blot detection of TERT in AGS cells after knockdown of SYVN1. (E) Control or SYVN1 knockdown AGS cells were incubated with cycloheximide (100 μg/mL) for the indicated times to determine the stability of the TERT protein in AGS cells. **(F)** Co-IP analysis of the interaction between CagA-EGFP and SYVN1-HA in HEK293T cells. **(G)** Western blot detection of TERT in AGS cells after the knockdown of SYVN1 and infected with CagA-HA lentivirus in AGS cells. **(H)** An endogenous ubiquitination assay shows the impact of CagA on the ubiquitination of TERT by SYVN1 in AGS cells. |

| **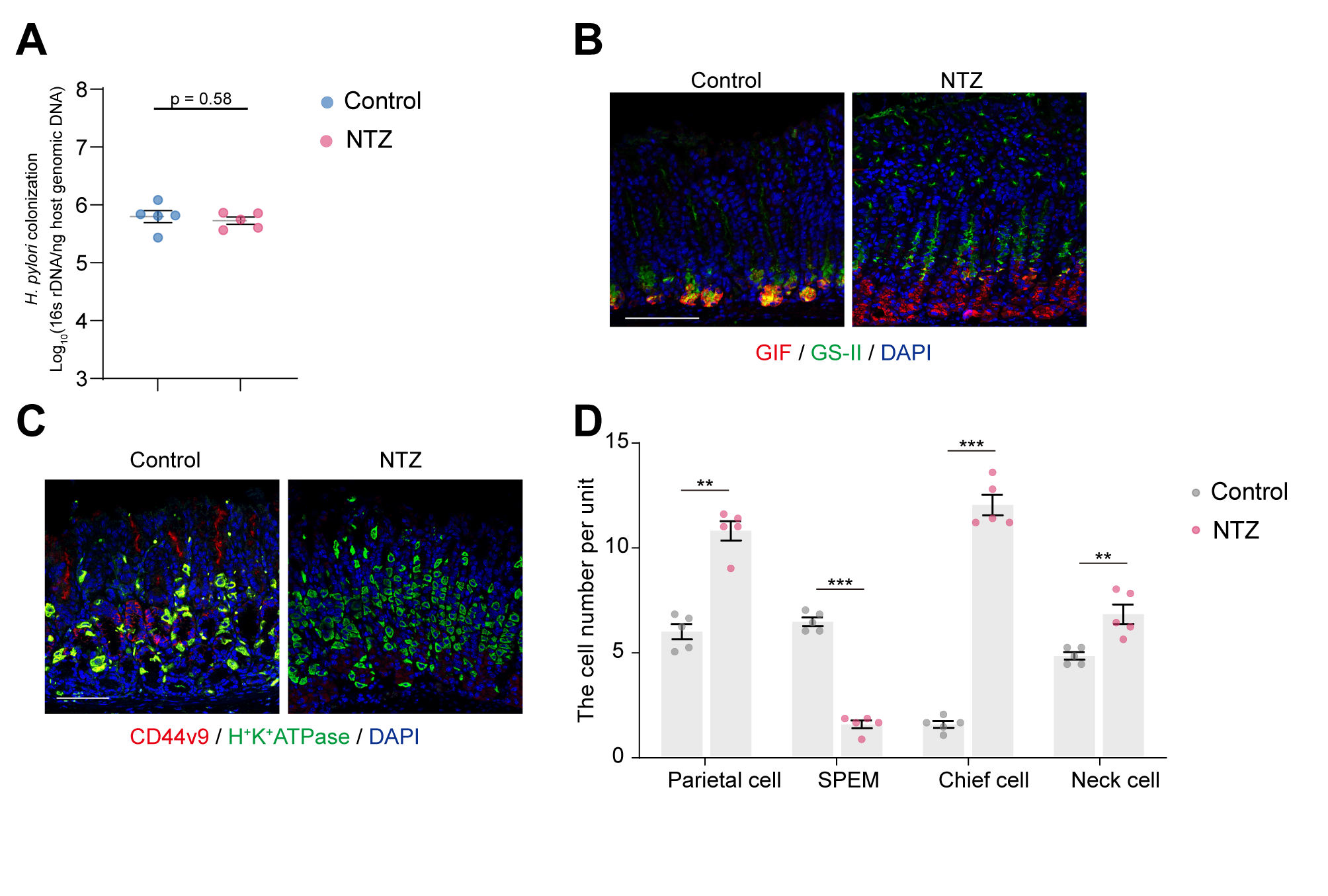** |
| --- |
| **Figure S9. NTZ does not affect the colonization of *H. pylori*. (A)** Probe-PCR for the colonization amount of *H. pylori* in gastric mucosa on control and NTZ group of *H. pylori*-infected mice (n=5). **(B)** Representative immunofluorescence staining of GIF (red color), and GS II (green color) on control and NTZ group of *H. pylori-*infected mice. Scale bar, 100 µm. **(C)** Representative immunofluorescence staining of CD44v9 (red color), and H^+^K^+^ATPase (green color) on control and NTZ group of *H. pylori-*infected mice. Scale bar, 100 µm. **(D)** Quantification of cell numbers per gland unit from panels B and C. Each group contains 5 mice. Each data point represents the mean number of each type of cell per gastric unit from ≥15 gastric units per mouse. |

| **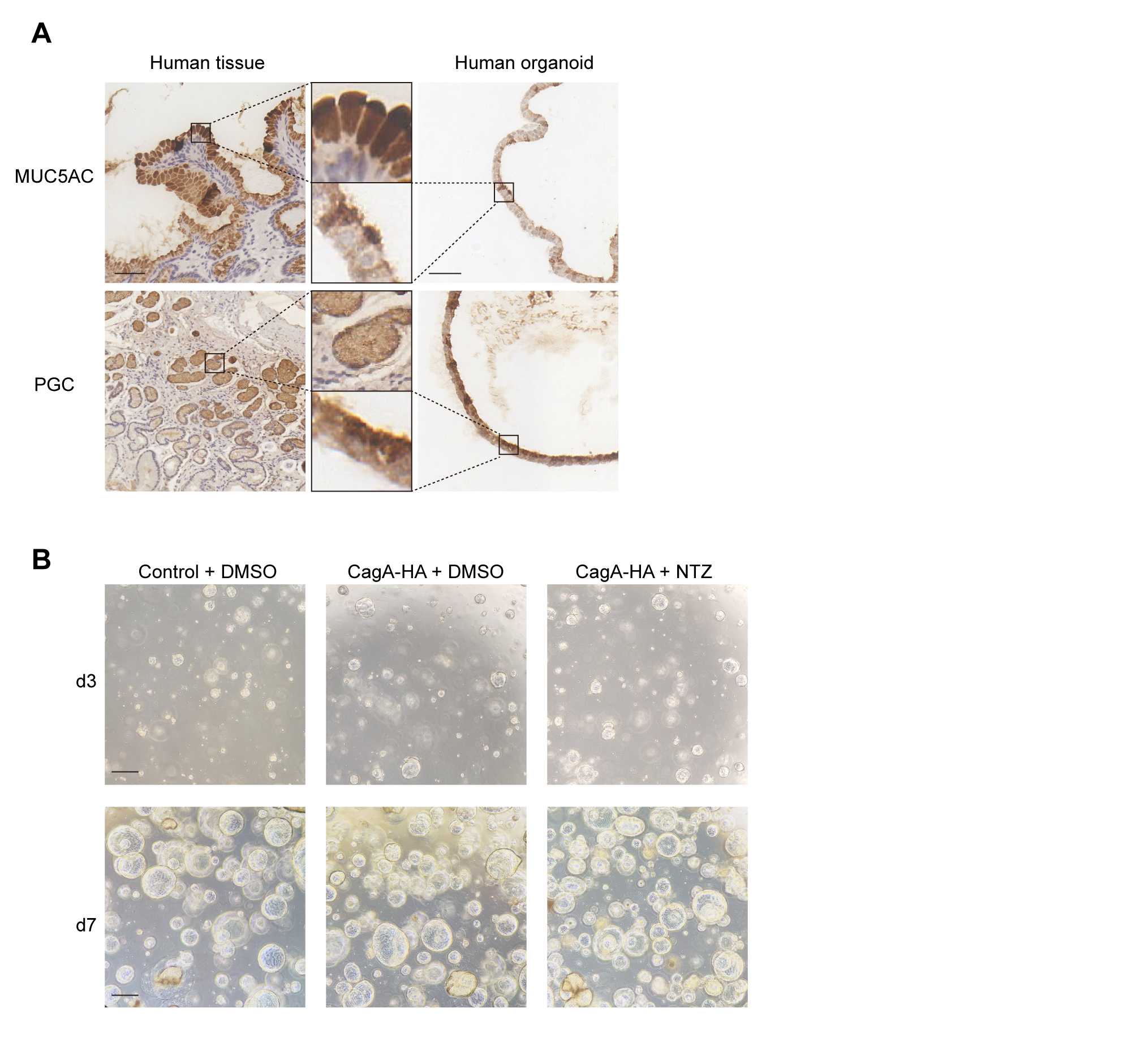** |
| --- |
| **Figure S10. Identification of major cell populations in human gastric organoids. (A)** The immunohistochemical staining intensity of MUC5AC and PGC in human gastric tissue and organoid. Scale bar, 50 µm. **(B)** Representative light microscopic view of organoid growth in the control, CagA-HA lentivirus infection group and NTZ treatment group. Scale bar, 100 µm. |

**Table S1.** Baseline characteristics of non-atrophic gastritis and intestinal metaplasia volunteers.

| Parameter | NAG | IM | P Value |
| --- | --- | --- | --- |
| Number | 105 | 115 |  |
| Age(y), min -max | 54.1, 28-75 | 58.8, 33-75 | ＜0.05 |
| Male/Female | 55/50 | 58/57 | 0.773 |

Inclusion Criteria: 25 to 75 years old; males and females are eligible; endoscopically diagnosed with intestinal metaplasia or chronic non-atrophic gastritis; voluntary participation in this study.

Exclusion Criteria: cardiovascular disease, coagulation disorders, metabolic disorders, and mental disorders (such as hypertension, diabetes, depression); use of antibiotics or probiotics within the past 3 months; history of alcoholism, smoking, or other tumors; pregnant or lactating women; History of esophageal or gastrointestinal surgery; other reasons that make painless endoscopy inappropriate; individuals who refuse to participate in this study.

**Table S2.** Primers used in this paper.

| Gene | Species | Primer | Sequence (5'- 3') |
| --- | --- | --- | --- |
| *Gapdh* | mouse | Forward | TGCACCACCAACTGCTTAG |
|  |  | Reverse | GGATGCAGGGATGATGTTC |
|  |  | Probe | CAGAAGACTGTGGATGGCCCT |
| *Gif* | mouse | Forward | GAAAAGTGGATCTGTGCTACTTGCT |
|  |  | Reverse | AGACAATAAGGCCCCAGGATG |
| *Clu* | mouse | Forward | CCAGCCTTTCTTTGAGATGA |
|  |  | Reverse | CTCCTGGCACTTTTCACACT |
| *Cftr* | mouse | Forward | TCGCTGGTTGCACAGTC |
|  |  | Reverse | TGCCTGAAGGGAGTCGT |
| *He4* | mouse | Forward | TGCCTGCCTGTCGCCTCTG |
|  |  | Reverse | TGTCCGCACAGTCCTTGTCCA |
| *Dmbt1* | mouse | Forward | CCAGGATGCAGATGTCG |
|  |  | Reverse | CACAGCTCCCAAGAGAATG |
| *Klf5* | mouse | Forward | CCGGAGACGATCTGAAACACG |
|  |  | Reverse | GTTGATGCTGTAAGGTATGCCT |
| *Ctnnb1* | mouse | Forward | ATGGAGCCGGACAGAAAAGC |
|  |  | Reverse | TGGGAGGTGTCAACATCTTCTT |
| *Cdh1* | mouse | Forward | CAGTTCCGAGGTCTACACCTT |
|  |  | Reverse | TGAATCGGGAGTCTTCCGAAAA |
| *Csnk1a1* | mouse | Forward | AAGGCCGAATTTATCGTCGGT |
|  |  | Reverse | ACTTCCTCGCCATTGGTGATG |
| *Pgc* | mouse | Forward | CCACCTACTACACTCAAGGGC |
|  |  | Reverse | AACTCCTGGTTAGGGACCTGG |
| *Pla2g1b* | mouse | Forward | CCGGGAGTGATCCCCTGAA |
|  |  | Reverse | ACCTGTCTAAGTCGTCCACTG |
| *Clps* | mouse | Forward | GAACAGTATGCAGTGTAAGAGCA |
|  |  | Reverse | GCAGATGCCATAGTTGGTGTTG |
| *Agr2* | mouse | Forward | GGACTCTCGGCCCAAACTAC |
|  |  | Reverse | GAATGACCATCAAGGGTCTGTT |
| *Car2* | mouse | Forward | GATAAAGCTGCGTCCAAGAGC |
|  |  | Reverse | GCATTGTCCTGAGAGTCATCAAA |
| *Cox4i1* | mouse | Forward | ATTGGCAAGAGAGCCATTTCTAC |
|  |  | Reverse | TGGGGAAAGCATAGTCTTCACT |
| *Fxyd3* | mouse | Forward | GGCTCATTTGTGCAGGGATTC |
|  |  | Reverse | TCTGTCTGAACTTGCATTTGCAT |
| *Chd7* | mouse | Forward | TTTTGGCGAGGATGGGAGTC |
|  |  | Reverse | CACGGGGTTTTCGGGGTAG |
| *Lrp5* | mouse | Forward | ACGTCCCGTAAGGTTCTCTTC |
|  |  | Reverse | GCCAGTAAATGTCGGAGTCTAC |
| *Trp53* | mouse | Forward | CCCCTGTCATCTTTTGTCCCT |
|  |  | Reverse | AGCTGGCAGAATAGCTTATTGAG |
| *Dvl1* | mouse | Forward | ATGGCGGAGACCAAAATCATC |
|  |  | Reverse | AACTTGGCATTGTCATCGAAGA |
| *Rac1* | mouse | Forward | ACGGAGCTGTTGGTAAAACCT |
|  |  | Reverse | AGACGGTGGGGATGTACTCTC |
| *Axin2* | mouse | Forward | ATGAGTAGCGCCGTGTTAGTG |
|  |  | Reverse | GGGCATAGGTTTGGTGGACT |
| *Il1b* | mouse | Forward | GAAATGCCACCTTTTGACAGTG |
|  |  | Reverse | TGGATGCTCTCATCAGGACAG |
| *Il1r1* | mouse | Forward | GTGCTACTGGGGCTCATTTGT |
|  |  | Reverse | GGAGTAAGAGGACACTTGCGAAT |
| *Il17a* | mouse | Forward | GGCCCTCAGACTACCTCAAC |
|  |  | Reverse | TCTCGACCCTGAAAGTGAAGG |
| *Il17ra* | mouse | Forward | AGTGTTTCCTCTACCCAGCAC |
|  |  | Reverse | GAAAACCGCCACCGCTTAC |
| *Il17rc* | mouse | Forward | TTCTGCGGTATTTGACTGTTTCG |
|  |  | Reverse | GTCCCGGACTTCAAGACCC |
| *ACTB* | human | Forward | TTCCTTCCTGGGCATGGAGTCC |
|  |  | Reverse | TGGCGTACAGGTCTTTGCGG |
| *TERT* | human | Forward | AGGTCAGGCAGCATCGG |
|  |  | Reverse | GCTCGGCCCTCTTTTCTCT |
| *CFTR* | human | Forward | CGAGAGACCATGCAGAGGTC |
|  |  | Reverse | TGTACTGCTTTGGTGACTTCCCC |
| *ATP4B* | human | Forward | AGGAGTTCCAGCGTTACTGC |
|  |  | Reverse | GGTCTTGGTAGTCCGGTGTG |
| *CDX1* | human | Forward | GGTGGCAGCGGTAAGACTC |
|  |  | Reverse | TGTAACGGCTGTAATGAAACTCC |
| *AXIN2* | human | Forward | CAACACCAGGCGGAACGAA |
|  |  | Reverse | GCCCAATAAGGAGTGTAAGGACT |
| *PGC* | human | Forward | TGGTCCTGGAGTCTTCTGGT |
|  |  | Reverse | GCTCCCGAATGATGCTGACT |
| *IL-8* | human | Forward | TTTTGCCAAGGAGTGCTAAAGA |
|  |  | Reverse | AACCCTCTGCACCCAGTTTTC |
| *16S rDNA* | *H. pylori* | Forward | TTTGTTAGAGAAGATAATGACGGTATCTAAC |
|  |  | Reverse | CATAGGATTTCACACCTGACTGACTATC |
|  |  | probe | CGTGCCAGCAGCCGCGGT |

**Table S3.** Antibodies used in this paper.

| **Antibodies** | **Brand** | **Code** | **Application** | **Dilution** |
| --- | --- | --- | --- | --- |
| lectin GS-II from  Griffonia simplicifolia, Alexa Fluor 647 conjugate | Invitrogen | Cat#L-32451 | IF | 1:500 |
| Mouse monoclonal anti-GIF | Santa cruz | Cat#sc-514524 | IF | 1:50 |
| Mouse monoclonal anti-H+/K+ ATPase β | Santa cruz | Cat#sc-374094 | IF | 1:25 |
| Rabbit monoclonal anti-PGC | Abcam | Cat#ab255826 | IF | 1:1000 |
| Mouse monoclonal anti-MUC5AC | Invitrogen | Cat#MA5-12178 | IHC | 1:150 |
| Rabbit monoclonal anti-TERT | Abcam | Cat#ab32020 | WB | 1:1000 |
| Mouse monoclonal anti-TERT | Invitrogen | Cat#MA5-16034 | IHC | 1:50 |
| Mouse monoclonal anti-CD44v9 | Abmart | Cat# M044707 | IF | 1:100 |
| Rabbit monoclonal anti-β-Catenin | CST | Cat#8480 | WB/IF/IHC | 1:1000/1:100/1:100 |
| Mouse monoclonal anti-β-TUBULIN | Beyotime | Cat#AT819 | WB | 1:1000 |
| Rabbit polyclonal anti-SYVN1 | Proteintech | Cat#13473-1-AP | WB | 1:3000 |
| Rabbit polyclonal anti-GFP | Proteintech | Cat#50430-2-AP | WB | 1:1000 |
| Rabbit monoclonal anti-FLAG | Proteintech | Cat#20543-1-AP | WB/IF | 1:5000/1:50 |
| Rabbit polyclonal anti-HA | Sigma–Aldrich | Cat#H6908 | WB | 1:3000 |
| Mouse monoclonal anti-HA | Proteintech | Cat#66006-lg | IF | 1:50 |

| 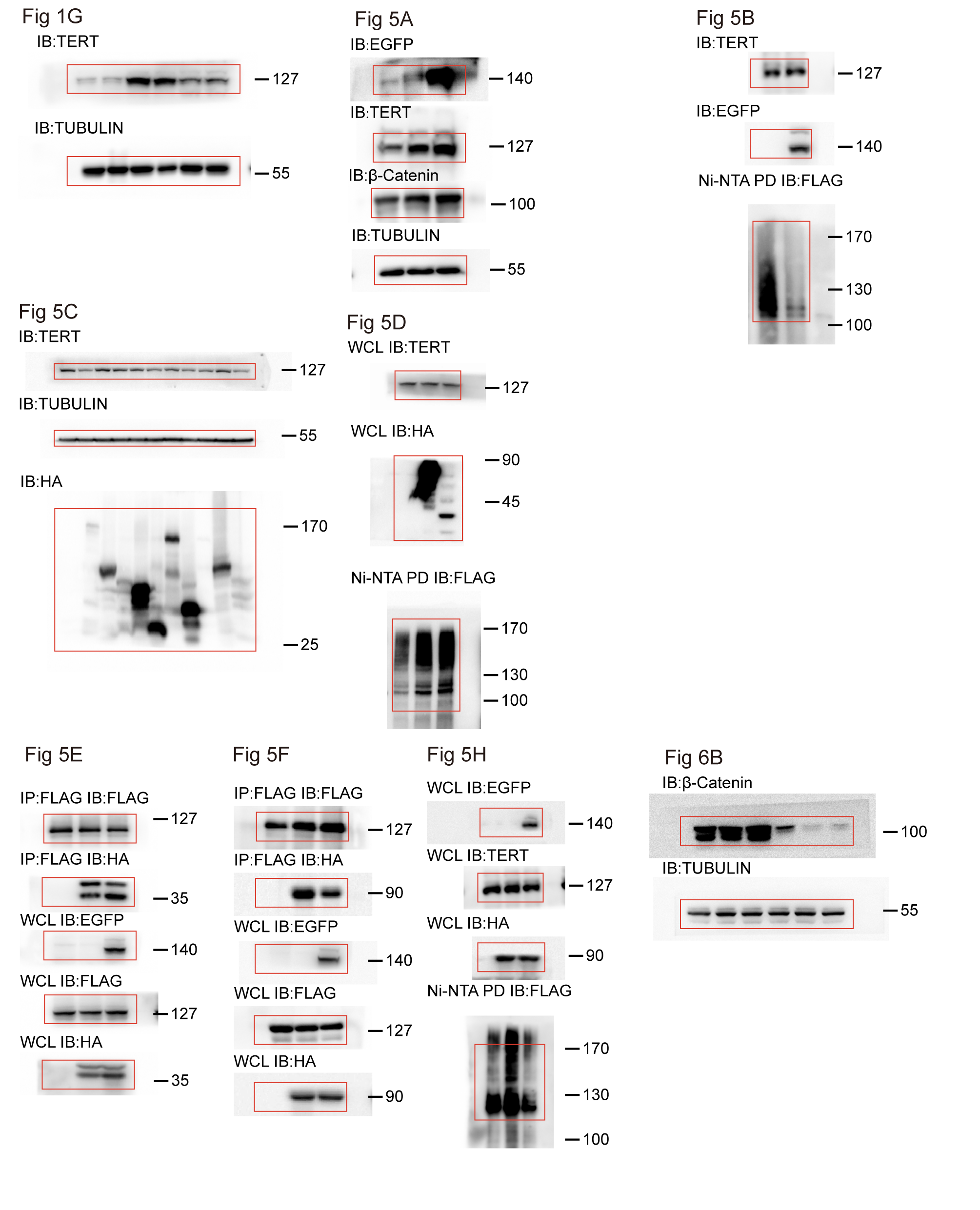 |
| --- |
| 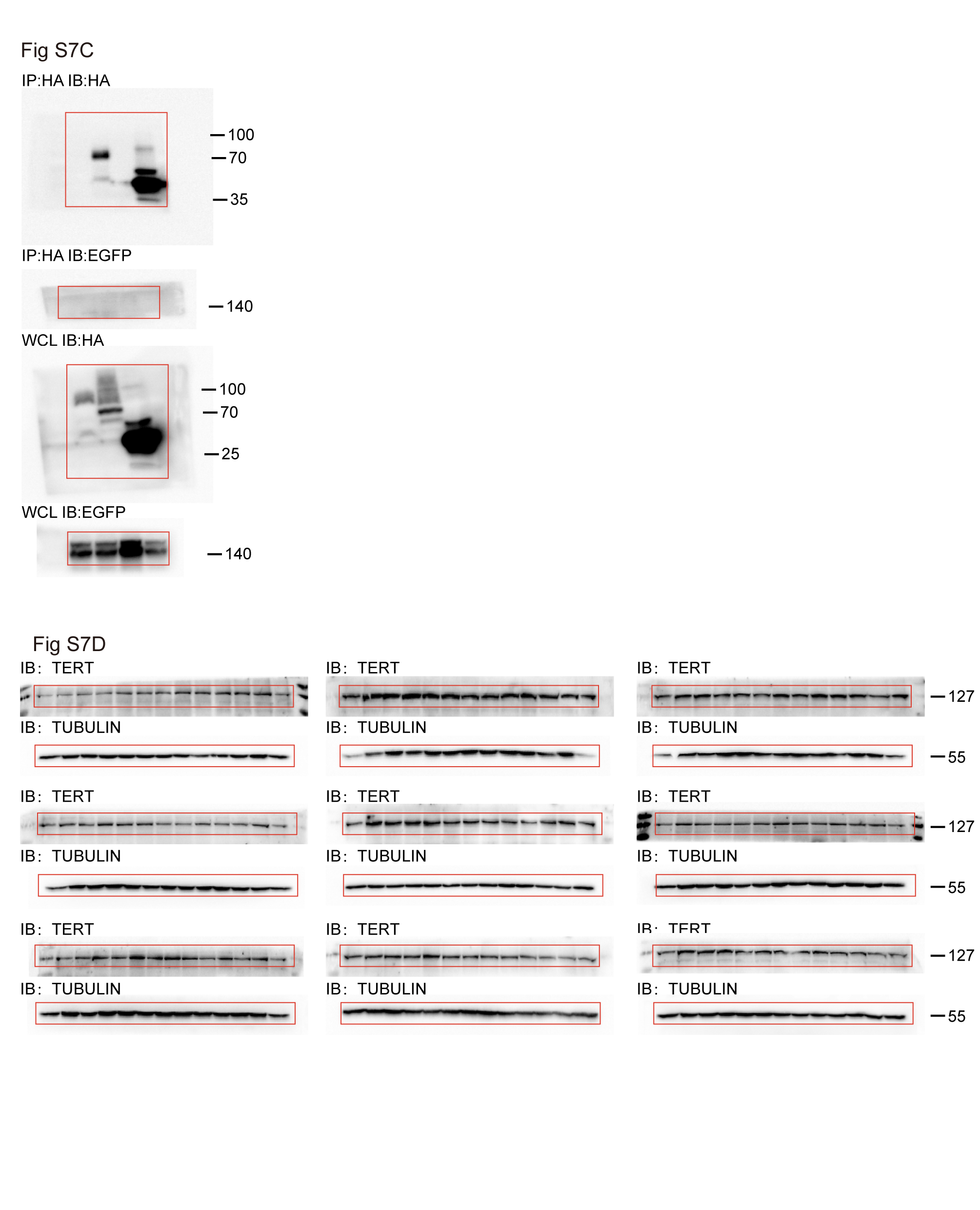 |


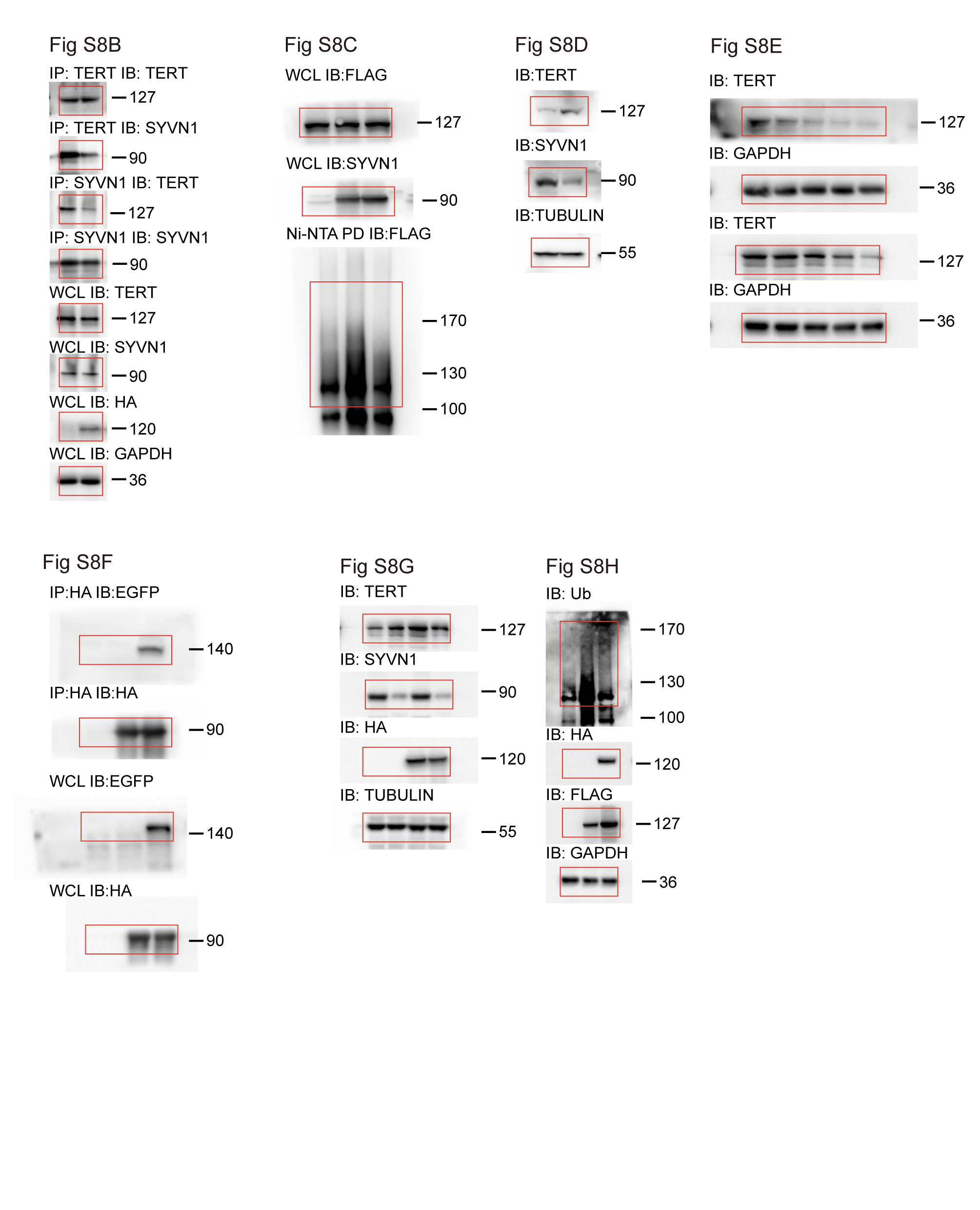

Supplement: Supplementary file 1 — Supporting Information [file ADVS-12-2401227-s001.doc]
